# Transforming multi-stakeholder engagement towards coproduction of optimized maternal, newborn, and child health and a resilient community health system in rural Ethiopia: A qualitative study

**DOI:** 10.1371/journal.pone.0330159

**Published:** 2025-08-26

**Authors:** Akalewold T. Gebremeskel, Ogochukwu Udenigwe, Josephine Etowa, Sanni Yaya

**Affiliations:** 1 School of International Development and Global Studies, University of Ottawa, Ottawa, Ontario, Canada; 2 School of Nursing, Faculty of Health Sciences University of Ottawa, Ottawa, Ontario, Canada; 3 The George Institute for Global Health, Imperial College London, London, United Kingdom; 4 University of Parakou, Faculty of Medicine, Parakou, Benin; Thammasat University, THAILAND

## Abstract

**Introduction:**

In Ethiopia, Maternal, Newborn, and Child Health (MNCH) outcomes have been improving, however, the current level of Maternal and under-five children mortality remains the highest in the world. Despite the rhetoric around the significance of multi-stakeholder engagement as a buzzword in development theories and polices to improve health and other development outcomes, there is limited evidence on how multi-stakeholders intersect and mutually reinforce each other toward the coproduction of improved MNCH outcomes and a resilient community health system. The aim of this manuscript is to examine barriers to and facilitators of coproduction in the context of multi-stakeholder engagement to optimize MNCH outcomes and a resilient community health system in rural Ethiopia.

**Methods:**

We conducted a qualitative case study in West Shewa Zone, rural Ethiopia. A purposive sampling technique was used to recruit participants. Data sources were two focus groups discussions with CHWs, twelve key informant interviews with multilevel public health policy actors, and a policy document review related to the CHW program to triangulate the finding. Thematic analysis of the qualitative data was conducted. Our study was informed by multiple theoretical frameworks including the World Health Organization’s building block framework, state- society synergy model to inform the research processes and analysis.

**Results:**

In the context of multi-stakeholder approach, our analysis revealed the multilevel barriers to and facilitators of coproduction in the community health landscape in rural Ethiopia. The major barriers of coproduction include lack of vertical and horizontal alignment, lack of continuum of and sustainable engagement practice,lack of systemic coordination platforms, and Inadequate coordination and implementation capacity. Major facilitators of coproduction include embedded integrated community health system, promising macro-level multi-stakeholder and community-level engagement and coproduction aspects*.*

**Conclusions:**

Our study reveals mixed policy and practice-related results, the current multi-stakeholder engagement is necessary but insufficient and fragmented to coproduce optimized MNCH outcomes and ensure a resilient health system in rural Ethiopia. Moving beyond the current multi-stakeholder engagement as a buzzword in health polices to practice through, embracing meaningful coproduction frameworks is fundamental while building on multi-stakeholder engagement efforts to optimize MNCH outcomes and a resilient community health system. A coproduction framework leverages the intersection and mutual reinforcement of multi-stakeholder synergy throughout the CHWs’ program cycle through shared power and joint assessment, planning, implementing, decision making and evaluating. Fostering effective multi-stakeholder engagement synergy requires balanced shared power, alignment to community priorities, systemic mapping, coordination and monitoring, and continuum and sustainability of engagement strategies. Beyond donor initiatives and a dependency approach, proactive health diplomacy strategies are also important to sustain the existing and attract new actors to realize sustainable positive health outcomes and a resilient community health policy and strategy.

## Introduction

In the last three decades, MNCH issues have gained significant global political economic attention, the responsibility for ensuring the right to health for all becomes not only with states and their obligations to their own people but also with the international community [1,2].This could be due in part to changes in global development thinking perspectives around reducing inequality since 1990. As a result, the global Maternal Mortality Ratio(MMR) saw a reduction of 34 percent between 2000–2020 and Under-Five Mortality Rate (U5MR) reduction of 59 percent between 1990 and 2021 [3]. However, despite the progress, sub-Saharan African (SSA) countries continue to have the highest MMR and U5MR occurrences in the world and this has become a key health system challenge in the region and persists as an important agenda of the Sustainable Development Goal (SDG) [4,5]). SDG # three aims to reduce MMR to less than 70 per 100,000 live births, reduce newborn mortality to at least 12 per 1,000 live births or lower in every country, and reduce the U5MR to at least 25 per 1,000 live births or lower in every country through multi-stakeholder engagement [2]. Therefore, to achieve the SDGs, Ethiopia must reduce neonatal deaths from 29 to 12 per 1000 live births and maternal deaths from 412 to 70 per 100,000 live births in an environment of increasingly constrained health resources.

The adoption of the Alma Ata Declaration in 1987 [6] of Primary Health Care has contributed to the acknowledgement of the fact that health determinants lie beyond the domain of the health sector and this has led to repeated calls for multi-stakeholder response [7]. Since the Alma-Ata Declaration, Ethiopia has made various attempts, including encouraging muti-stakeholders engagement, to improve MNCH outcomes and achieve Universal Health Coverage (UHC) [8]. Ethiopia introduced an ambitious nation-wide community health program in 2003 with plans to improve access to primary health promotion and essential MNCH for disadvantaged rural communities [9,10] The current level of better health outcomes including MNCH is attributed to the CHWs program. Since the launch of the program, Ethiopia has reduced maternal and child mortality by half. The CHWs program is run by CHWs who are situated at the lower levels of the hierarchy of the health system. Ethiopia has a top to down, vertical decentralized health system along with a decentralized regional (provincial) political structure. The Federal Ministry of Health (MoH) is mandated to formulate national policies, strategies and standard [8]. MoH works with different levels of the health system’s administrative hierarchies. The highest to the lowest hierarchies include MoH[8], regional health bureaus, zonal health offices and district health offices. District health offices are the bottom or micro level of health administration to facilitate primary healthcare service provision including the community based Health extension Program(HEP) [8].

However, due to the fact that the Ethiopian health system is in a fragile state [9,11], the national wide CHW programs continued to experience multiple challenges to enhance better health outcomes [9,12–16]. The supply side health program has overwhelmingly failed to address the quantity and quality of health workers, an under-resourced health system, poor infrastructural development, and social determinates of health [9,15,16]. Even though health spending is increasing, Ethiopia is one of the several countries in Africa which falls short of the international health expenditure benchmarks [17,18]. The government of Ethiopia allocates about 7.8% of its national annual budget to health which is still far from the Abuja commitment of 15% [17]. The skilled health workforce is constrained and the health sector has been heavily dependent on fragmented external funding [9,10,19]. The ongoing COVID-19 pandemic is amplifying the problems of an already overwhelmingly fragile health system [20,21].

In Ethiopia, like in many other Low- and Middle-Income Countries (LMICs), in the demand side the rural community health program has a complex socio-political economy. There are wide gaps and inequality in the availability and utilization of MNCH services in rural and urban areas of the country [22]. Higher inequalities of MNCH outcomes continue to exist between rural and urban residents, and among different regions of Ethiopia [22]. Inequality in MNCH outcomes is increasing over time, the demand side factors of MNCH outcomes inequality in Ethiopia includes low-economic status, illiteracy, lack of health and others infrastructural development [22]. The most disadvantaged in society includes women, rural residents, the uneducated and unemployed [22,23].The deprived areas and disadvantaged communities have difficulties in accessing means of prevention and adequate MNCH services due to weak health systems and fragile logistics.

In the last three decades, MNCH issues have gained significant attention, in Ethiopia and internationally, multiple efforts have been made to address health system challenges and improve MNCH[(1–3]. During this time, multi-stakeholder initiatives are ubiquitous in health polices as a key strategy to address national and international SDGs. The 2030 agenda necessitate a whole-of-society strategy, SDG 17 recognizes multi-stakeholder partnerships as important vehicles for mobilizing and sharing knowledge, expertise, technologies, and financial resources to support the achievement of the SDGs in all countries. SDGs calls for the meaningful and active participation of stakeholders at all levels to realize progress on the SDGs and to ensure that no one is left behind [24]. According to the SDGs, all stakeholders including governments, citizens, Non-Governmental Organizations (NGOs), Civil Society Organizations (CSO), academia and the private sector all have roles to play in contributing to SDGs. Multi-stakeholder partnership is increasingly highlighted in Ethiopia’s health policy reforms [8,25] to address the health system challenges and improve health outcomes including MNCH. In this article, we follow the widely used understanding of ‘stakeholders’ as individuals, groups, or organizations that affect or are affected by organizational activities like policy making, development, implementation, or management, as described by Freeman(1984) [26].

Better health outcomes are not a product manufactured by the healthcare system alone, but requires the coordinated engagement of actors beyond the health professionals [27]. Building on the theory of Evans(1997)’s state-society synergy and Ostrom’s co-production [28,29], we argue that it is becoming more common and crucial for more stakeholders to be involved in producing and improving policy outcomes. Co-production is prevalent and becoming the ‘emerging paradigm’ in development discourse and practice [30]. In emerging political economies where governments have limited capacity to provide public service, there is often no alternative to co-production [31]. Co-production goes beyond traditional consumer participation models, the production and delivery of optimum services is difficult without the active engagement of the beneficiaries [29]. In co-production approaches, citizens are not the passive targets or beneficiaries of government activities, but become vital elements in their success or failure [32]. Building on Elinor Ostrom’s Political Theory and Policy Analysis [29],coproduction can be defined as the integration of multiple actors in the production of public services [32].

Multiple efforts have been made to enhance multi-stakeholder engagement in health program development from the local to international community. Multi sectoral collaboration ‘Health in All Policies’ is a systematic approach which emphasises a broad multi-sectoral method for any national health plans, and to address all the determinants of health [8]. Community engagement like Health/Women Development Army were introduced to promote HEWs programs and the services [9,33]. This entails collective actions by wide-ranging actors outside the health sector, such as education, women affairs, agriculture, and water, within the framework of health determinants. The large portion of health finance comes from donors and development partners in the form of loans and donations from all over the world (46.8%), the Ethiopian Government is the second largest source (16.5%), followed by out-of-pocket payments (35.8%), and others (0.9%) [34,35]. In many countries, the COVID-19 pandemic underscored the need for multi-stakeholders to prevent and control the virus and reduce its impact in collaboration with government.

Despite the rhetoric around multi-stakeholder engagement as a buzzword in development theories and polices [8,25], evidence is limited on how the muti-stakeholders interact and mutually reinforce each other towards coproduction and improved MNCH and a resilient community health system in Ethiopia and beyond. Multi-stakeholder engagement encompasses various actors, such as governments, the private sector, civil society, academia, and international organizations. Unlike public-private partnerships, which mainly focus on formal collaboration between state and private entities, multistakeholder engagements tackle complex challenges that necessitate diverse expertise and resources. These engagements prioritize the active involvement and contributions of all stakeholders, including civil society and the public [36]. There are bodies of literature addressing the need and practice of multisectoral engagement for finance and resource mobilization more broadly [37]. The existing evidence focuses on individual actors [38–41] without considering the comprehensive synergy among multiple stakeholders in the context of CHWs program and MNCH in rural Ethiopia. In addition, the existing evidence on stakeholder engagement largely focuses on patient and stakeholders’ involvement in health research and guideline development but does not provide guidance on how multi-stakeholder engagement should be aligned, interact, and mutually reinforced in health policy practice and evaluation [42–44].

The aim of this manuscript is to examine barriers to and facilitators of coproduction in the context of multi-stakeholder engagement toward the optimized MNCH outcomes and a resilient community health system in rural Ethiopia.

### Theoretical framework

Our study was guided by different theoretical frameworks to inform the research processes and analysis. First, we used the socio-ecological framework to inform the description of the multilevel determinants of the CHWs’ program effectiveness [45,46**]**. The socio-ecological framework posits that factors at various levels uniquely and jointly contribute to health interventions namely, individual level, interpersonal level, community level, and system level factors [45,46**]**. Second, our study was informed by the WHO’s Health Systems Framework, a leading structure of discourse on health systems building. The framework comprises six operational building blocks—service delivery, health workforce, information, medical products and technologies, financing, and leadership and governance, it however falls short in including macrolevel community context and engagement [46]. Third, we used the Synergetic model: synergetic model shifts the focus from state-private business coproduction to ideas that involve state-society organization cooperation [28]. State-society synergy is a framework used in analyzing the relational behavioral pattern between the state and non-state and market actors. We used a synergistic model based on the works of WHO’s Health Systems Framework [28], Judith Tendler’s [47] concept of blurred public-private boundaries, and Elinor Ostrom’s (1996) vision of “coproduction” [29].

**Fig 1:**
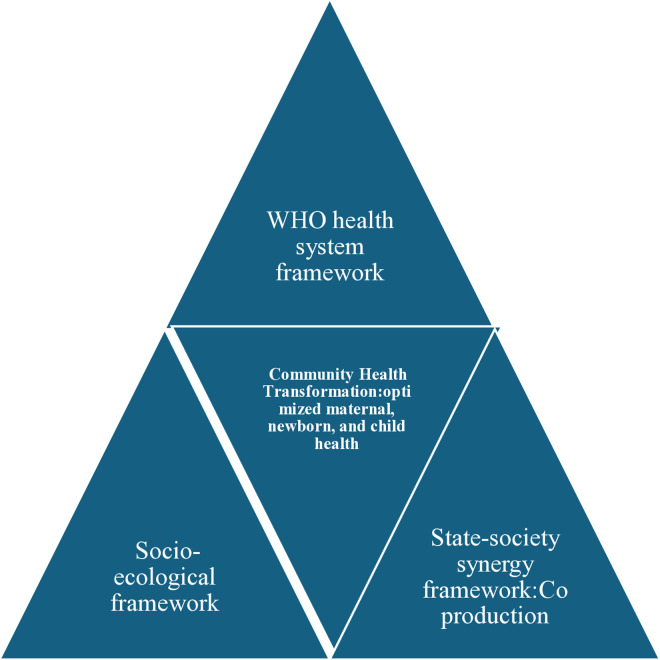
Multidimensional and integrated theoretical approach for the community health transformation framework (Developed by the author, ATG).

Synergy is defined by Evans as a win-win relationship, which can be achieved by “Complementarity‟ which is premised on mutually supportive relations between public and private spheres and “Embeddedness” whereby close relations between public and private citizens are forged and maintained [28]. State–society synergy asserts that active government and mobilized communities can enhance each other’s developmental efforts [28]. Ostrom (1996) emphasizes the fact that, synergy cannot be achieved if public officials and citizens continue to see a great divide between them [29]. Furthermore, we draw on Kingdon’s multiple streams framework [48]: Kingdon’s approach provides the conceptual framework for the analysis of the three streams – problems, policies, and politics. Kingdon argues that policy change occurs when there is adequate attention to a problem (the problem stream), a policy solution has been clearly articulated and reached consensus (the policy stream), and there is political will to adopt this policy (the politics stream). When all three streams converge, a policy window opens, representing an opportunity for advocates of proposals to push attention to their special problems [48]. Fig 1 presents an integrated framework combining the socio-ecological model, the WHO Health Systems Framework, and a synergistic multiple-streams policy window perspective.This approach emphasizes context-based multisectoral engagement to strengthen community-based health systems and improve maternal, newborn, and child health (MNCH) outcomes(see Fig 1).

Our findings were reported based on the Consolidated Criteria for Reporting Qualitative Research (COREQ) (see S1 File).

## Methods

This study is a qualitative case study using document review, Focus group Discussions (FGDs)) and Key Informant Interviews (KII). Qualitative case study is a research methodology that helps in the exploration of a phenomenon within some context. Desai and Potter [49] stressed that development study requires the use of a wide range of research methods: the mix of methods enables the different techniques and their results to be compared against each other, allowing judgements to be made as to which method (or combination of methods) is the most appropriate for any particular purpose. In a case study, a real time phenomenon is explored within its naturally occurring context, with the aim of answering the “how” and “why” questions [50,51]. Yin proposes that a case study inquiry should rely on multiple sources of evidence, because data source triangulation allows the researchers to have a trustworthy groundwork for the findings and the contribution of knowledge [51]. According to George and Bennett [52], case studies enable deep contextual understanding and they have potential for achieving high conceptual validity; case studies are a useful means to closely examine the hypothesized role of causal mechanisms in the context of individual cases. The move from single-case to paired comparison offers a balanced combination of descriptive depth and analytical challenge that progressively declines as more cases are added [53].

### Research setting

Ethiopia is one of the most populous countries in Africa with 109 million inhabitants, which is second to Nigeria. The World Bank [54] classifies Ethiopia as a low-income country, but it is also one of the fastest growing economies in SSA. It still has a largely rural population, above 80% of the population lives in rural areas, and 12–14% of the total population are pastoralists or agro-pastoralists [55]. Since the adoption of the 1994 ethnic based constitution, the government of Ethiopia has been structured in the form of a federal system of government. The country is administratively divided into eleven ethnic based regional states (provinces) and two chartered cities that are administered separately from states [56]. This study was conducted in Oromia regional state, which is the largest of the eleven Ethiopian states, both in terms of population and landmass [56].

Ethiopia has a top-down decentralized model for the delivery of healthcare along political structures. The health sector is structured into a three-tier system: tertiary care, provided at specialized hospitals; secondary care, provided at general hospitals with inpatient and ambulatory services; and primary healthcare (primary hospitals, health centers and HEWs post) [25]. Each primary healthcare unit serves approximately 25,000 people. The CHWs/HEP program is the primary health system component at the community level that reaches the rural majority communities by recruiting and training paid community based HEWs [10].

In this research, the nested nature of the case study encompasses two distinct populations including HEWs/CHWs (16 FGD participant), and subnational and national public health policy actors (12 KII participant). These populations are spread over three geographical areas with respect to the scope of their work: West Shewa Zone CHWs, Oromia Region (subnational) and Ethiopia (national) public health policy actors.

The FGD data was collected from HEWs in West Shewa Zone. West Shewa is one of the zones of Oromiya region, the zone has 23 districts and is located to the west of Addis Ababa (Capital city of Ethiopia). The zone has a human population of 2,058,676, of which 1,028,501 are males and 1,030,175 are females. In this Zone, in 2019, 84 per cent of the population lived in rural areas [57]. Currently, the zone has 8 hospitals, 92 health centers and 528 CHW posts (each post has an average of 2 HEWs). The CHW program and health posts are administered and funded under the public health system. This location was chosen for research because despite the progress in other parts of the country, the West Shewa zone is still underperforming when it comes to MNCH outcomes.

### Participants selection

#### FGD participants.

All participants, CHWs/HEWs, had to be adults ranging from the ages of 21 and who had been residents of the district (work site). The participants must have had one-year experience working as a CHWs in MNCH before COVID-19; three and more years of CHW/MNCH work experience in rural area; over one year of HEWs national training, must be full time employee and be salaried. The study did not exclude HEWs/CHWs by their gender or sex.

#### KII participants.

Key informants were recruited based on their known involvement in the policy process leading to the planning and the implementation of CHWs/ MNCH program. Snowball sampling was used to recruit participants whereby participants were suggested and recruited by other participants [58].

The principal investigator (AG) estimated the saturation of data between 10–12 interviews and 2 focus groups and 6–8 individuals based on previous similar approaches [59].

### Participant recruitment

#### FGD recruitment.

Using purposive sampling, AG worked with the Oromia Regional Health Bureau (ORHB) and zone health administration in an attempt to select the two districts. Then AG contacted and worked with the district health office to recruit potential FGD participants to send the recruitment poster to potential participants via the districts’ regular means of communication, telegram, email and announcements during review meetings.

#### KII recruitment.

The principal investigator, AG, worked with the MoH, ORHB, zone health administration to select the first potential participant in the KII. AG started by sending an invitation email with information about the study and the KII recruitment poster. The source of the referral (the referee) was confidential. The potential invited participant had the right to accept or decline the invitation, and this response was confidential too.

We used a first-come-first-served basis for enrolment. Prior to participating in this study, participants were asked to provide their free and informed consent by signing a consent form.

### Data collection

#### Research instrument.

The data collection was organized in three parts: FGDs, IDIs and document review. AG collected the data using semi structured FGD guides developed in English and translated to Oromo language and KII guides developed in English and translated to Amharic and Oromo language. The guides were carefully crafted following the theoretical frameworks and relevant literatures to include neutral, non-biased, and non-leading questions to avoid influencing participant responses. AG is fluent in the local language. Based on their agreement, AG contacted participants for their convenient time and place to sign the consent form and then conducted the FGD and KII. AG acted as the facilitator to recruit participant, ensure confidentiality, and distribute and collect consent forms.

At the beginning of each FGD and KII, AG briefly described the purpose of the study to the participants. All FGDs and 11 KII were conducted face-to-face, 1 KII was conducted using zoom. After consent was given, each FGD and KII was recorded(audio). The focus group sessions lasted between 80–90 minutes and the KII lasted between 60–90 minutes. A research assistant was assigned to take notes during the FGDs after a brief training about the research ethics and process by AG. The research assistant was fluent in local Oromo language, possesses a BSc degree and research-related experience.

As a token of appreciation, all FGD and eight KII participants were compensated with a small honorarium, for their time and effort and to cover their lunch related expenses. In the context of COVID-19, participants adhered to public health guidelines including wearing masks.

Most of the data collection was conducted face-to-face in Afan Oromo and Amharic, based on participants language preference. Two FGDs were conducted with CHWs working in two different districts/ *Weredas* (*Adea Berga and Ejere*) of West Shewa Zone. In-depth semi-structured KIIs were conducted with twelve key informants from national and subnational level health systems.

Data collection took place at different locations depending on participants. The FGDs were conducted in a rented hotel hall while KII with policy makers were conducted in convenient locations for participants such as their offices.

We conducted a document review to enrich the finding from the FGD and KII, and to understand the context and operation of the HEWs/CHWs program. Policy documents, including national/regional strategies and plans were considered to represent the major priorities for CHWs led MNCH. The relevant and available document were selected and accessed through the recommendation of the key informants working in National and subnational HEP/MNCH program in Ethiopia.

### Data analysis

We followed Braun and Clarke’s [60] six step thematic analysis. The WHO building block framework and SEM guided the data analysis and interpretation of the qualitative data sets. FGD and the KII were audio-recorded document and AG transcribed verbatim. Verbatim transcription was done by listening to audio-recorded materials. The two FGD and four KII in Oromo language, and eight KII in Amharic language were transcribed into word documents. Then, the transcribed document in both languages were translated to English language. Data were also cross-checked by listening to the original recording while reading along in English. A literal translation (word-for-word) was employed to maintain the original responses of participants and help readers grasp their mindset. The translation process included back translation, which involved translating the data from the source language (Oromo and Amharic languages to the target language (English) and then reverting it back to the source to resolve any ambiguities or inconsistencies [61]

The transcript content was coded manually to ensure the quality, accuracy and any discrepancies of coding transcript. Codes were developed after an initial review of the transcripts. The transcript content was analyzed inductively to get familiar with the data. First, after familiarization to the transcript AG & OU coded one transcript from each, FGD and KII, they coded individually and compared their codes. After debriefing and consensus, the transcript was coded accordingly. After debriefing, JE and SY randomly chosen and compared to check alignment or discrepancies of the coding of the transcript. Emerged themes were based on an iterative process of inductive and deductive approaches. Deductive approaches were drawn from existing literature and the systematic review phase and the study theoretical frameworks. In inductive approaches, themes emerged from the data and were not be forced to fit into preconceived categories [62]. The analyses used triangulated data from multiple sources and multilevel government and non-government health policy experts and frontline CHWs, and document analysis. Given the small size of the data, we decided to code manually without employing any qualitative data analysis software.

This study takes a fully theory-informed inductive study [63] design In this approach, the theoretical framework informs the research questions, the approach to research, the concepts that underpin the study design, the choice of methodology, data collection, interactions with study participants, analysis processes, and the conclusions drawn. By acknowledging that reality is subjective and varies from person to person and by centering the participants as holders of knowledge, this study draws on constructivist ontology and epistemology [64].

### Policy documents

Policy documents were significant to the study, and were used mainly from a realist perspective [65], that is, as a means to understanding the CHWs/HEP policy/ program in the context of rural Ethiopia. Hence, policy documents were essential in providing background information to the study and in defining the questions and trajectories that were pursued in the FGD and KII. The analysis was also enriched by useful insights from the policy document. Using thematic analysis, we sought a document that mentions the major themes that emerged from the KII and FGD, based on inductive and deductive approaches. Major Health policy documents used in this study includes second Health Sector Transformation Plan (HSTP-II) [8], A roadmap for optimizing the Ethiopian HEP(2020−2035)[25 and more, see the list of policy documents (see S2 File).

### Trustworthiness

Trustworthiness, a qualitative validation criteria, was applied in this study in line with established guidelines [51]. Therefore, the researcher’s inherent bias was controlled partly by being self-aware and giving the participants the opportunity to generate data based on their own perceptions. We enhanced the trustworthiness of the study’s findings by using different techniques recommended by renowned qualitative researchers including member checks, peer review, and triangulating the data by using multiple sources of data to confirm emerging codes and themes to serve as a data validation strategy and help to establish the credibility of the study findings[(66,67].

### Ethical considerations

The research proposal was carefully evaluated, and the ethics of the study subsequently reviewed and approved by two respective institutions, the University of Ottawa Research Ethics Board, ethics clearance certificate (see S3 File-Ethics File Number S-06-22-8072). And the Ethiopian Public Health Institute, Institutional Review Board (EPHI-RIB) certificate of approval (protocol number: see S4 File-EPHI-IRB-462–2022, minute No:109). In addition, to strengthen the importance of the study, support letters were received from School of International Development and Global Studies, University of Ottawa and form the study area, Ethiopian MoH, ORHB and West Shewa health office. Data collection period was between September 15, 2022 to end of November28, 2022.

Participants gave their free and informed consent to be enrolled in the study. Participants provided written informed consent prior to participating in this study. They were also informed that once they chose to participate, they could withdraw at any time or chose not to answer any questions, to which there would be no negative consequences.

Written informed consent were collected by the principal investigator (AG). AG immediately stored the softy copy/electronic files on a password protected computer that is in a locked office at home and on a secure network. Then signed consent forms were teared up and thrown in a secure garbage bin. All our research participants were (women and men) adults over age 21. We did not collect data from minors.

### Researcher reflexivity and positionality statement

To enable any audience of qualitative studies to evaluate the validity of conclusions extrapolated from data, researchers should, as part of the study, neutralize or bracket their biases by stating them explicitly to the full extent possible [67]. AG, OU, JE and SY have extensive experience as global health experts and have multiple publications and work on MNCH and related policy and programs in SSA including Ethiopia. AG has more than eight years in community health program management in Ethiopia and worked in different contexts of health programs implementation and evaluation in Ethiopia.

## Results

### Characteristics of participants

The sociodemographic characteristics of Focus Groups Discussions (FGDs) participants is summarized on Table (see S -5) In FGD, a total of 16 Female health extension workers (HEWs/CHWs) from two district of west Shewa Zone. All the FGD participants, HEWs/CHWs, are female, and majority 15(93.75%) of them are in the age range of 25–40. The highest level of education or training that they have completed: college diploma 12(75%) and over one year HEWs training 4(25%). Participants’ total work experience as a HEW (in years), six and above 14(87.5%) and four to five years 2(12.5%). For all the participants, walking was their most commonly used mode of transportation during field work/ outreach activities. The average distance between their work site and field work/outreach site in kilometer: above 5 km, 9(56.25); 3 km to 5 km 5(31.25%); and less than 3 km 2(12.5%).

A total of twelve public health experts were recruited to participate in the Key Informant Interviews (KIIs). Eight participants (66.6%) were recruited from three levels of government health structures (three participants from MoH, two participants from ORHB, and three participants from West Shewa and two district level health offices). Four (33.3%) participants were recruited from NGOs (two from the National level and two from the regional level. In terms of gender, four (33.3%) women and eight men (66.6%) participated. Most of the participants have post graduate level (MSc) educational background in health and related study programs and they have extensive (more than 10 years) public health related experiences in different levels of responsibilities.

Power dynamics and their expression in multi-stakeholder relationships are crucial factors, as the values, intentions, and interactions of both individuals and organizations largely influence the connections between actors [68]]. This research acknowledges the potential power imbalances stemming from those who have the authority to make and influence decisions across different contexts. This topic is further examined in the discussion section of the paper.

###  Barriers to multistakeholder engagement

In the context of multi-stakeholder engagement, our analysis revealed the multilevel barriers to and facilitators of coproduction in the community health landscape. The major manifestations of lack of coproduction includes lack of vertical and horizontal alignment, lack of continuum of and sustainable engagement practice, lack of systemic coordination platforms, and Inadequate coordination and implementation capacity. Major facilitators of multi-stakeholder engagement towards coproduction include embedded integrated community health system, promising macro-level multi-stakeholder and community-level engagement and coproduction aspects.

Based on the detailed analysis, we summarize and present the major finding below. In this paper multi-stakeholder refers to the organizational activities of multi-stakeholder acting in the Ethiopian health system’s land scape including community and their institutions, multi-sectoral line ministries, CSO and NGOs, Donors and development partners, Private sector, academia and research institutes. Also, Macro level refers to National level context, Meso-level refers to regional level context, and Micro level refers to Zonal and district context.

The subthemes include: The challenge of alignment (vertical structures and horizontal relationships), Lack of continuum of and sustainable practice, Lack of systemic coordination mechanism, and Inadequate coordination and implementation capacity.

#### 1. The challenge of alignment (vertical structures and horizontal relationships).

In the context of multi-stakeholder engagement, our finding highlights alignment-related challenges at the CSOs/NGOs and community level, they are characterized as vertical and horizontal fragmentation related challenges and a lack of system aligned holistic support. Throughout the multilevel health system structures, health actors are not linked to the needs and expectations of grass root communities.

At the COS/ NGOs level, implementing partners submit their intervention proposal and receive approval from respective national or regional level bureaus. Then, implementing partners appear at their area of implementation without prior consultation, prior communication, pre assessment or joint priority seating and planning with the direct beneficiary community and district level health offices.

*“Most implementing partner project agreements are made at the Federal or Oromiya regional level. They come to us for actual practice/implantation, this is a transparency gap, we have no prior information about the project and the duration of the project. There is also, a transparency gap among some partners who are not clear on what they are doing, and for how long they are doing it. I don’t mean all.” (KII 9, Zonal level health MNCH expert). “Stakeholders integration is not uniform in some districts, there is strong and functional collaboration. In some distract the collaboration is moderate and unfortunately null in some areas.” (KII 8, MoH, National HEWs/CHWs expert).*

There is a gap of vertical alignment to the local context or problems and expectations of the community. Government has the ultimate power to initiate health policy and practice, top to down community engagement is problematic in ensuring community trust and meaningful community engagement (CE). A top-to-down professional-led CE approach without a balanced grass root voice/perspective to the top alignment and integration into the community-based institution, culture, and norms is a challenge to ensure ownership and sustainability.

“From *the beginning, I don’t think the community participation approach was context-specific and volunteer-based. If this was the case, we wouldn’t have such an interruption. Community participation is still high on other issues…however, their participation in health, education, and other government program is not functional. The approach is not on volunteer bases, there is some political or administrative force behind it. The community has its own organization like ‘idir’or ‘iqub’ they are very committed to those.”(FGD participant, From Adea Berga District)*

*“The community engagement approach has been a top-down prescription. It is only the implementer who knows the success and the challenge. End-users have limited participation. So now we are trying to kind of reverse that. When we do the community engagement, how is it functioning first? What is their challenge? What should they hold? What do you contribute? It was functioning in that manner by understanding what they were saying.” (KII 7, MoH, Primary health system expert).*

Using a predetermined issue, government and the implementing partners both focus on the donors’ interest, not on the local interest and problem. The current interventions are partner-driven and top to down stretched, partners are not initiating their project from the zone or community.

*“From my practical experience…CSOs/NGOs focus on the donors’ interest, not on the local interest. NGOs/ partners are not initiating their project from the region, the zone, or the community; they approach the government with a predetermined proposal. In one of my previous implementing partner organizations, we directly started working on the project with CHWs but when the implementing organization phased out the project, the activities initiated by the project were stopped. This is because despite working with CHWs/HEWs, there was no integration with the existing local government structure in starting and co- designing the project. There was no co-planning with the district health office and health facilities did not own the project because of lack of co-creation; this is a co-creation gap.” (KII 12 Regional NGO, Public health Expert)*

Beyond the top-to-down prescribed interventions, there is often a culture and language barrier between the community and the implementing partner (NGOs/CSOs).

*“The NGOs have the intention to work for the community and in the community. But they have limited capacity, they don’t have enough knowledge of the culture of the project area, and they don’t speak the language of that community. This is a big challenge, often there was no communication with the community, there was no community integration to understand and address the problem and to support and own the project. NGOs should have appropriate human power with the knowledge of the values and the language of that community to be more effective.* **(***KII 5, Regional health bureau, Primary health expert)*

Lack of system aligned holistic support: Arguably, holisticness of the program could make the Ethiopian community health system unique, however, there is a lack of a holistic community health program strengthening support system to optimize CHWs accessibility and MNCH outcomes.

*“There is an inconsistency between CSO actors and programs. For example, the HEWs program requires holistic intervention. However, CSO/NGO actors come with a specific program. They could come just for immunization they have the challenges to support the whole program. The government does not want it, and that is one of the challenges. When a partner comes without system-supporting capacity, it doesn’t strengthen the program.” (KII-1, MoH, National level primary health program expert)*

Lack of inclusiveness in multilevel structures and multiple aspects of the program, instead of incorporating the implementing partners as a stakeholder in every aspect of the CHWs cycle, often there is externalization of stakeholders.

*“They cannot have real unity and so they cannot bring change. The health sector shouldn’t see the NGOs as external bodies, now there is a progress, there is a progress of seeing each as a part of the same system in terms of building trust among each other to improve the benefit of the community. The health sector should accept them, as they came to work on the health sector task.* **(***KII 5, Regional health bureau, Primary health expert)*

#### 2. Lack of continuum of and sustainable practice.

Our analysis shows that the current multi-stakeholder practice and community-based health intervention face challenges related to a lack of continuum and sustainability. The term “continuum” refers to the continuity of meaningful engagement, synergy, or partnership in the community health program cycle: joint planning, implementation, and evaluation to enhance coproduction.

*“I have over then years of experience in government health sector and partners, I know many NGOs and projects, there is a big gap in health interventions project ownership and sustainability. Some of the initiatives are always backsliding. There is lack of co-creation, joint project planning, implementation, monitoring, reviewing and the like. If there is cocreation, the stakeholders feel ownership and take care for it as they have co-designed and implemented. Then the project will sustain whether the partner exist or not.” (KII 12 Regional NGO, Public health Expert)*

The existing state-multi-stakeholder synergy lacks continuum of engagement in the community health program cycle.

*“The community engagement strategy is not well organized. There is some community participation, for times when the higher-level leaders give attention or direction it would be hot. It is just like a campaign, sometimes strong and sometimes very low. The overall approach of community engagement is not planned and regular. The community is suspicious and generally lacks trust [in the health system]. When you invite the community for different health issues, they assume that there is an attached political issue, so they are not willing to participate. When sometimes the community is engaged on health issues, there are also political issues, so the community doesn’t what this, the community do not engage.” KII 10, Regional NGO, Program manger*

*“…, but the main challenge is there is no regular meeting and monitoring mechanism. I think they should have regular planning, monitoring, and review mechanisms, at least every three months. This can give a chance to see the progress and the challenge, then to address them in collaboration. (KII 4, District health office, HEP experts)*

Often the community engagement platforms are donor-driven, project-based, and short-term result oriented, these pose a challenge to the sustainability of projects. The strategy is not functioning in a sustainable way, there are always interruptions. Short term result-oriented projects are problematic.

*“Nowadays, the community structure is not functioning well as it was intended. It is characterized by considerable dropouts or interruptions. Now, it is interrupted and there are dropouts as the people developed negative attitudes toward such a structure. Sometimes the way of organization and participation is attached to political activities and orientation.” (FGD participant, From Adea Berga District).*

Despite multi-stakeholder engagement in the health program, fragmented resource allocation for health intervention is also a big challenge to ensure health intervention sustainability.

*“Some organizations come with limited budget, so it is important to use that limited resource on specific activates instead of multidirectional activities.” (KII 9, Zonal level health MNCH expert).*

*“Okay, compared to other sectors, there is a large number of NGOs working on maternal health and family health, reproductive health. But this is not comparable to the level of problem and demand, they are few in number, and the problem of the society is large and severe. Even though the NGO’s budget amount is not comparable, there is a gap between the level of the problem and the budget, we need more support internally and internationally.”* **(***KII 5, Regional health bureau, Primary health expert)*

Lack of self-esteem, donors’ dependency syndrome is also a challenge to ensure sustainable community health system. The “dependency syndrome” is an attitude and belief that the Ethiopian government cannot address the health system challenge without the initiatives of donors’ and development partners’ support.

*“I don’t think that there is a simple way to move out of aid dependency in a short time***.** *Yes, various donors help the health sector program. The health sector is one of the beneficiaries of SDG resources pooled from different donors. So, the health sector uses more of donors’ budget. So, as you said there should be a way to enhance domestic resource mobilization. Increasing the export item to improve the economy of the country.” (KII 8, MoH, National HEWs/CHWs expert).*

*“Most of the health budgets come from partners, partners were covering the budget for capacity-building activities, and partners also had a significant role in terms of supply and logistics.” (KII 9, Zonal level health MNCH expert).*

“*If the government of Ethiopia is not able to receive the donors support, I don’t think the government can do it alone. Majority of the health sector programs are from the donors support. The budget allocated by the government is very limited. Health sector relays on the support from donors and partners.” (KII 12 Regional NGO, Public health Expert)*

*I don’t think the government alone can resolve the health system challenge. Even the government officials say this. Even, after some projects are completed and handed over to the government, there is a time when they area not able to sustain the project.” (KII 11, National NGO, HEWs program M&E)*

Inadequate health diplomacy: Despite the efforts of the government to create a conduce environment, the CSO amendment in 2019, and the ambitious HEWs/CHWs Optimization launch(25), the efforts to advocate for and attract the multiple national and international public health actors is limited. Majority of the public health workers have limited knowledge about the CSO proclamation amendment.

*“In my perspective, the CSO proclamation amendment alone would not improve the CSOs engagement, but the reverse is happening. CSO proclamation alone can not bring significant change, [long laugh], I am not good in politics, So, I think the international diplomacy is wakened, maybe this is due to the current political instability or lack of capacity. I was among those who were so hopeful on the improvement. The interest of USA is not clear during this political crisis. Donors have no interest due to the political instability.” (KII 12 Regional NGO, Public health Expert)*

*“Since the amendment of the CSO proclamation in 2019, I didn’t see any significant changes.”(KII 10, Regional NGO, Program manger)*

*“There was one plane, one budget, and one report, to coordinate the partner’s budget under one plane, budget, and governance system. I said earlier, the partners are leaving and so it is not working as planned. When there is an available budget, using under one doesn’t have any problem, rather it enhances budget effectiveness. However, in practice, it is not progressing currently.” (KII 9, Zonal level health MNCH expert)*

In the current context, initiatives are coming from partners, not from the government.

*“I think the government should create a conducive environment. The government should engage partners as a sector. The government should recognize the sector and invite them. But the current practice is that partners with interest go to the government. It means that the initiative is from the CSOs. But should come from the government too so that different sectors can participate as one of the development actors.” (KII 12 Regional NGO, Public health Expert)*

*“When the project is phased out some NGOs are properly transferring the project to the government to ensure sustainability. In some cases, there is a cooperation gap. If the Health sector is not participating in the processes there is no proper handover during the project phase-out time. This is not good.* ***” (*** *KII 5, Regional health bureau, Primary health expert)*

The volatile security and political issues are exacerbating the challenge of continuum and sustainability of engagement challenge.

*“For example, the conflict that started two years ago has set back a lot of things. It has had a big effect…. there are certain gaps that need to be addressed.” (KII 7, MoH, Primary health system expert).*

*“The health sector is particularly affected by the political crisis. Funds have declined significantly. The health extension program is particularly affected. That’s where most of the problems come from. Related to that, not only the public sector but also other sectors have been affected.” (KII 7, MoH, Primary health system expert).*

For more perspective, See more quotes on S6 File

#### 3. Lack of systemic coordination platforms.

Our findings illustrate that the existing CSOs/NGOs-state synergy is not effectively functioning due to a lack of adequate coordination platforms. Lack of system coordination refers to the inadequate systemic management capacity for optimum functioning of the process of community health program intervention. The multilevel macro-level, meso-level and micro-level barriers include inadequate coordination capacity, inadequate capacity to monitor the program, manage the limited resource, inadequate health diplomacy, volatile security and political issues.

#### 4. Inadequate coordination and implementation capacity.

There is a challenge of inadequate coordination from macro-level, to meso- and micro-level and on both CSO/NGOs and the government’s side. CHWs are overburdened to facilitate and coordinate the multi-stakeholder engagement. Two HEWs serve about five thousand people with 16/18 HEP packages with limited knowledge, skill and motivation. CHWs are are also working on other government tasks, outside health sector activities.

“*There is a leadership role challenge from the Ministry of health to the CHWs, grass root level. There is a leadership, management and commitment problem to transform the policy on paper to action.” (KII 12 Regional NGO, Public health Expert)*

*“There is a problem on the side of civil society. There is a lack of coordination in the civil society organization, it would be good if things here and there are coordinated together.” (KII 2, National level NGO, Program Director)*

*“There is annual joint planning, the” Woreda Base plan”. But there is a challenge with the follow-up. There is no system of follow-up on how the resources were utilized. Theoretically, the plan is expected to be one. The allocated budget should be known or announced. But there is no monitoring system or officer. Report and idea exchange stop here. It is the partner’s responsibility to submit a report, there is no prior notice or request from the government side.” KII 10, Regional NGO, Program manger)*

“*There is a human resource challenge. Two HEWs serve about five thousand people this is because of the financial challenge. They could have done if you have more health workers. It is very challenging for 2 HEWs to provide services for 3000-5000 people. The have limited knowledge, skill and motivation. They do all this by walking multiple kilometers to provide home to home service. If reducing the package is not possible, then increase the number of health workers. They don’t have sufficient working and living situation. More training opportunities should be facilitated for them. ’KII-12 regional NGO expert.” (KII 12 Regional NGO, Public health Expert)*

*“Yes, the HEP packages are 18 and are many given their number, sometimes one some times two, About 7 out of the 18 packages focus on maternal and child health. Of course, overpacking will slow down the performance quality and quantity. In addition to those packages, they are also working on other government task, out of health sector activities. For example, health extensions are part of local cabinet. It also brings problems; it creates additional workload. I think the solution could be increasing the number of the HEWs and separating MNCH as a specific program. Given the number of activities in MNCH, separation of the program is very important.” (KII 9, Zonal level health MNCH expert)*

The current coordination platform is not uniform and functional up to the district level. There is a problem with organizing and planning the available resources, and there is a resource shortage at the service site.

*“I can’t say out loud that the coordination platform is uniform and functional up to the district level. It is not possible to say that 100 percent will be implemented as agreed here, because the lower structure may be influenced by partners’ interests. Partners may lobby by providing incentives for lower-level officials, incentive can twist. As you said, people who are incentivized in connection with poverty and incentives can twist some things, so I can’t say that it has been fully implemented.” (KII 7, MoH, Primary health system expert).*

Participants indicated that not only is the resource allocation and support for coordinated efforts inefficient, there is inefficient capacity to manage the limited resource. Duplication has been a glaring manifestation of muti-stakeholders coordination barrier in Ethiopia.

*“…most partners do not have an office at the woreda level, except for some local NGOs. The most common problem raised by the district is on resource allocation, support is insufficient. They say that the demand is high, and our gap is high, but the support is less. In some districts, they can work very friendly and considerate. Multisectoral integration is not uniform in some districts there is strong and functional collaboration, in some distract the collaboration is moderate and unfortunately null. There is a strong Woreda-based planning engagement and ISS facility-level integrated supportive supervision every year.” (KII 8, MoH, National HEWs/CHWs expert).*

*“I think that this is very clear, I don’t believe that the government will work independently. Despite the multiple work done so far with the support of NGOs, there is still a gap. I don’t think it can be done by govt alone. I think for the future, there should be proper resource management approach, there is a moment the resources are wasted unnecessarily**..”* *(KII 10, Regional NGO, Program manger)*

“*Despite the effort towards changing the situation, NGOs have been free to select their project implementation site and project activities. There have been duplication issues, some districts have no NGO support, lack of budget, project phaseout time/ contract period being over, and maybe the country’s current situation.* **(***KII 5, Regional health bureau, Primary health expert)*

For more perspective, See more quotes on S6 File

### Enablers of multi-stakeholder engagement

The subthemes include Embedded integrated community health system; promising macro-level multi-stakeholder engagement and coproduction aspects; promising community-level engagement and coproduction aspects.

#### 1. Embedded integrated community health system.

The Ethiopian community health program is nation wide and embedded into the community and the health system. The CHWs/ HEP program is the extension of national primary health system with a holistic multi-package of services (18 Packages including MNCH components).

As indicated in the macro-level policy documents, like HSTP-II [8], a roadmap for optimizing the Ethiopian HEP(2020–2035)(25), it is designed to address the health access needs and rights of the disadvantaged rural majority population using community as a service user and key stakeholder.

In contrast to other countries, the CHWs program, which is designed based on specific disease or preprogram, the Ethiopian community health program is an integrated mix of multiple-packages. The CHWs program is designed to provide an integrated multi-package of services under a single program coordination, majority of the components HEP are related to MNCH and it is tailored to the needs of mothers and children in disadvantaged contexts. Participants indicated that female HEWs were preferred because majority of the components of health extension program are related to MNCH

*“The health extension program is an effective option, and this is not only to our country, but the world has witnessed and learned from it. What makes ours special is the fact that the one in other countries more of barter driven and non-institutionalized with the health system, but ours is aligned with the government health system with paid staff to maintain the system well. In addition, what makes it different from other countries is that other countries mostly work on a single initiative through their community health program; Or it could be a family planning area or RMNCH (Reproductive, Maternal, Newborn, Child Health) area or disease prevention or other programs.” (KII 7, MoH, Primary health system expert).*

The CHWs/HEP program is a conducive landscape for multi-stakeholder engagement to address the various challenges of the health access and determinates.

“**Health extension programs are not sector specific in nature. Another part of the government sector is seeking the engagement of the private sector. We think we have engaged the government sector very well. Other sectors also need to engage and collaborate.” (*KII 7, MoH, Primary health system expert).*

#### For more perspective, See more quotes on S6 File. 2. Promising macro-level multi-stakeholder engagement platform and coproduction aspects.

Our findings reveal that efforts have been made to engage multi-stakeholders through policies and strategies in community health program landscapes. Perspectives on the macro-level multi-stakeholder engagement platform indicates the need for better accountability programs and community participation.

There are macro-level multi-stakeholder related policies and strategies to enhance engagement and stakeholders’ partnership in the community health program. These include the revised Civil Societies Proclamation No. 1113/2019, One Plane, one budget, and one report strategy. The revised Civil Societies Proclamation No. 1113/2019 (called CSP/2019), opens the space for CSOs to carry out a vital role and make meaningful contributions by lifting the disabling rules of the previous law. The CSP/2019 abolished the 10% rule on funding enabling CSOs to raise funds from any lawful source without requesting approval from the Agency, the law gives a flexibility of administration and program activities and transferring project equipment to another project after phaseout.

*” The Proclamation before 2019 had many restrictions, and those restrictions were lifted to bring in and use donor and foreign aid better. Moreover, domestic civil society organizations were in a situation where they were missing out on budgeting, so I think that by adjusting them, they will develop their capacity and improve the health sector and other sectors as well.” (KII 7, MoH, Primary health system expert).*

The MoH One Plane, one budget, and one report approach were introduced to guide the harmonization and alignment of stakeholders’ funding to health sector plans and health outcomes.

*“The One plane, one budget and one report is important. It enhances cooperation during planning and performance and monitoring. There is such approach in the health sector level, but in practice there is a gap. There is a great expert turn over, after a short time the expert who have planned would not there during planning, they may leave the position or the sector. New staff may have limited experience. One budget will help to save the resource or avoid resource wastage, with one budget it is possible to address multiple tasks.”* **(***KII 5, Regional health bureau, Primary health expert)*

There is a macro level promising initiatives of joint planning, implementation and evaluation initiatives and joint technical working group and a steering committee in every level.

*“As a ministry of health, we involve all levels from woreda to the ministry of health on one plan, one budget, and one report. This is one part of the efforts of the MoH to engage the grass root, going down to the district and coordinate.” (KII 8, MoH, National HEWs/CHWs expert).*

*“There are some initiatives, Joint project inception, planning, implementation. NGOS/partners are working with government. Some partners are employing/ assigning secondments, these are like ambassadors. In Oromia health bureau there are about 10-12 staff assigned as a seconded to provides technical support and share their organization’s experience excellence, play a liaising role, ensure their brand. Like a bridge between the government and parties. Seconded is not enough, there is a gap in joint problem identification, project selection and project cocreation.” (KII 12 Regional NGO, Public health Expert)*

Our finding shows the existing and emerging multi level stakeholders have a great interest to work on reproductive health including MNCH. Participants acknowledged the support from donors such as the UNICEF but contended that joint planning, joint implementation and joint evaluation between the government and partner organizations is crucial.

*“Okay, compared to other sectors, there is a large number of NGOs working on maternal health and family health, reproductive health. But this is not comparable to the level of problem and demand, they are few in number, and the problem of the society is large and severe. Though the NGO’s budget amount is not comparable, there is a gap between the level of the problem and the budget, we need more support internally and internationally…The government alone could not deliver the MNCH. Because most of the previous achievements are due to partners’ effort and support.”* **(***KII 5, Regional health bureau, Primary health expert).*

For more perspective, See more quotes on S6 File

#### 3. Promising community-level engagement and coproduction aspects.

All participants stressed that community engagement (CE) is crucial for the success of CHW program in rural Ethiopia. There are different platforms of CE in Ethiopia. These include engaging indigenous institutions like ‘Idir’ ‘Ikub’, Traditional Birth Attendants and CHWs program-based groups like Women development groups. In Ethiopia, health policies and strategies like HSTP-II [8] and a roadmap for optimizing the Ethiopian HEP(2020–2035) [25] community engagement is one of the strategic approaches to ensure CHWs program for rural and remote populations.

“*The role of the community is big. Every aspect of our work needs community engagement whether vaccination, maternity health, or communicable disease. Mainly, we were using the Women Development Army, we also use key community leaders and religious leaders then they inform the community in their respective…. The government must give more attention to the program, but the community can do what ever they can as long as there is transparent discussion/ communication on how to improve and contribute in the health system. I can say that there is no problem from the community. Currently, there is no specific project implemented government alone.” (KII 4, District health office, HEP experts)*

Women’s groups are the most active and functional platforms of MNCH service promotion and demand creation, “as clients and change agents.”

“*Mostly, the women’s group structure is the most functional one. Women have ‘One to Five’ structure which is a group formed taking into to account neighborhood. They are selected and organized form the community to promote MNCH services like when pregnant women should visit the health facility for ANC and their children vaccination.” (FGD participant, From Adea Berga District)*

*“More of the Health Extension Program is related to women and children; efforts have been made to organize women to work as clients and change agents. At that time, I was also engaged, so I tried to make them aware. When the document was prepared, an effort was made to understand from them their challenge and how to solve it.” (KII 7, MoH, Primary health system expert).*

Aspects of community engagement are potential source of resource mobilization and include in cash and in-kind resource mobilization. The community has been showing a great potential to support the health system, there is nothing that government can implement without the active participation of the community. The community is significantly engaging through contributing cash to buy an ambulance, building a maternity waiting home near the health centers and contributing crops to feed pregnant women while at health facility for delivery.

*“In my perspective, there is nothing that government can implement without the active participation of the community. The communities’ role in HEWs program is not easy, while the government was supplying the nails, and copers, the community was engaging in providing their labor, wood, and superstructure. Many health posts (60%) were built by community participation.”(KII 8, MoH, National HEWs/CHWs expert).*

“**In addition to the ambulance supplied by the government, the community also made a demand, so they donated a lot of cash to buy an ambulance and make it available, and in terms of building a maternity waiting home near the health centers, the community contributed a lot. But when I say this, I do not mean that there are no challenges.” (*KII 7, MoH, Primary health system expert).*

“*Our communities are actively participating. They contribute 100 ETB for Ambulance purchase/ maintenance, they are contributing crops for pregnant women, they are renovating the health post and building the fence of the health post through their own material and labor. They are even contributing their resources in cash or/in kind to support to construct the health center.” (KII 4, District health office, HEP experts)*

Community Based Health Insurance (CBI), pooling funds to offset the cost of healthcare, is becoming the emphasis of the Ethiopian health policy and practice [8,25]. There is a growing interest on strengthening CBI to ensure sustainable health services. Participants anticipate that CBI will improve the health-seeking behavior and strengthen the health financing challenge of the informal sector, including rural communities who base their livelihood on agriculture. However, there were concerns that the CBI may be ineffective.

*“We are promoting CBHI at scale rather than making them pay from their own pockets while going through the health check and the health extension program. CBHI is now compulsory; already been ratified. As of this year, all regions are implemented that way. For this, except for those that are done by free will, those who can are benefiting by paying. A client goes to a health post, then a health center, then a hospital.” (KII 7, MoH, Primary health system expert)*

Participants explained that coproduction, which they identified as knowledge sharing, enhanced ownership and sustainability enhances the community health system and MNCH:

*“Co production is important, because knowledge is every where, I have it you have it, and it also exists every where. If we bring it together to produce it would enhance ownership, or you could not blame some one. If the project by NGOs/ governments starts from the community through co-creation all respective stakeholders to identify the problem and to design the solution together we will not fail as pervious or should not be give us a blinking result (the result sometimes seen and disappear sooner). It would give us a community owned and sustainable result to improve our country’s health system, this is the way I think.” (KII 12 Regional NGO, Public health Expert)*

Effective community engagement should start from the bottom grass root level. A crucial aspect of meaningful community engagement in the rural Ethiopia community health system is the community’s ownership of health programs. Building on the existing indigenous social structures in the community and ‘the whole community engagement approach’ is crucial to ensure inclusiveness of the community participation and to enhance the synergy.

*“Last year I worked on one project as a consultant in “Borena area.” I can not tell you how much I was pleased to see such co creation approach. We called all the respective stakeholders from the HEWs, from the community rep, religion institution, community leaders, from health sector, from health, women, social affairs. We came up with very genuine gap/ problem identification with the stakeholders, this is our problem and we need this to be solved for us. Now the project is started in a very good way.” (KII 12 Regional NGO, Public health Expert)*

*“To improve the engagement of the community we should organize the community into different groups based on their situation. Then, we have to create favorable conditions for participation. Inviting the community during planning and incorporating their suggestion in the planning, instead of top to down planning. I think, this has a better output. Working with the community can help to prevent child vaccination dropout. Community engagement should be maintained.” (FGD participant, From Adea Berga District)*

For more perspective, See more quotes on S6 File

## Discussion

Over the last two decades, the health outcomes in Ethiopia, including MNCH outcomes, have been improving, however the current highest level of negative MNCH outcomes is unacceptable. Despite the muti-stakeholder engagement in the country wide CHWs program, there is limited evidence on how they interact and mutually reinforce each other towards the coproduction of optimized health outcomes. We conducted a qualitative case study to examine barriers to and facilitators of multi-stakeholder engagement toward the coproduction of optimized MNCH outcomes and a resilient community health system in rural Ethiopia. In the context of multi-stakeholder engagement, our analysis reveals the multilevel barriers to and facilitators of coproduction in the community health landscape. The major manifestations of lack of coproduction includes lack of vertical and horizontal alignment, lack of continuum of and sustainable engagement practice, and lack of systemic coordination platforms. Major facilitators of multi-stakeholder engagement towards coproduction include embedded integrated community health system, promising macro-level multi-stakeholder and community-level engagement and coproduction aspects.

In the context of multi-stakeholder engagement, our discussion focusses on the barriers to coproduce improved health outcomes in community health system in Ethiopian. First, according to our finding the current Ethiopian multi-stakeholder approach lacks adequate vertical and horizontal alignment to the need of the community, there is a limited effort to incorporate communities voice through prior need assessment to define the problem and plan the solution jointly. The existing engagement often meant inviting the beneficiary community and local/ district implemented to be informed on new plans, or to solicit their support for decisions that had already been made; it remained far from putting community and local stakeholders in the driver’s seat. This could be due to the power imbalance, the macro level government has the ultimate power to initiate health policy to be practiced in a top to down fashion [8,25].

The top to down engagement approach is problematic in ensuring meaningful synergy of coproduction. CHWs programme needs to be multidimensional rather than vertical and they should rely on all stakeholders [69]. The collaboration should influence public services outcomes, it should go beyond collecting input from users [70]. In Malawi, multi-stakeholders felt that due to lack of top to down and bottom to up balanced stakeholder engagement, the prevailing engagement approach is dominated by tokenistic consultation. Multi-stakeholder have no power to influence health policy-making to better meet their health need [71]. There may be challenges such as lack of commitment by policy-makers and decision-makers to engage the multi-stakeholder [19,72]. Since experts from the public and NGOs have more resource available, often control and initiate these processes, define the scope of activities, and consequently serve their interest and lead to further power inequality [73]. Genuine power-sharing, partnerships, bidirectional learning, incorporating the voice and agency of beneficiary communities determines the effectiveness of community engagement [74]. In Ethiopia the community engagement framework and strategies should be guided by a framework that describes how empowered public institutions and polices disregard power to citizens, and how levels of citizen agency, control, and power can be increased [75,76]. When designing and implementing community health development programmes, community ownership, leadership. adequate resources, continual monitoring tools and motivation are essential considerations through the continuum of working with people to help them identify and address key problems affecting their community [77].

Second, our finding shows that the existing multi-stakeholder approach in Ethiopia’s community health program is fragmented and not well aligned to the holistic community health program system strengthening. While multi-stakeholders are engaging in different levels of health program intervention, they use selective approaches, disease-specific interventions, and their effects may undermine progress towards long-term impact like building resilient health system. Fragmented community health programming is a challenge for community health program effectiveness in SSA [46]. As a result, in LMICs like Ethiopia, the increased spending has not achieved fundamental shifts in health system [74]. An inclusive framework is important to synergize the multi-stakeholder efforts to coproduce a resilient community health system, beyond short-term outcome approach [46,78].

Third, according to our analysis the existing state-multi-stakeholder synergy lacks continuum and sustainable engagement in the community health program cycle; it is characterized by considerable dropouts or interruptions and some of the initiatives are always backsliding. According to our analysis, there is lack of continuum of co-creation, joint project planning, implementation, monitoring, reviewing and the like. Despite more engagement of stakeholders during COVID-19, it was only for short time and was an ad hoc strategy. It seems the focus of the current engagement is achieving the short-term project results through periodic demand creation and health promotion activities [79]. It is very crucial to engage stakeholder throughout the health policy cycle [71,80]. In Malawi, the perceived barriers to stakeholders’ meaningful engagement in the health policy process includes tokenistic involvement, stakeholder hierarchy; mutual distrust; preferred stakeholders and no culture of engagement [81]. Coproduction gives opportunity for multiple actors and perspectives to address complex continuum and sustainable challenges through joint effort.

Fourth, our analysis illustrates that in the context of multi stakeholder engagement, there is a challenge of fragmented coordination from macro-level, to meso- and micro-level. There is no adequate and uniform monitoring system up to the district level to organize the program and the available resources. Lack of systemic coordination is becoming a key health system challenge, driven by increasing fragmentation [82,83]. Also, our finding shows there is inefficient capacity to manage the limited resources; CHWs are overburdened to facilitate and coordinate the multi-stakeholder engagement. This could be associated to the insufficient manpower at lower level, two HEWs serve over three thousand people with 18 HEP packages with limited facilities. “Mottos like ‘universal health coverage’ and ‘no one left behind’ cannot be achieved without training and deploying more community health workers” (p#2) [84]. A systematic review that summarized CHWs’ perceived workload across low- and middle-income countries supports the observation that CHWs endure a considerable workload [85]. This study suggests that a heavy workload can adversely affect their productivity and significantly harm both their physical and mental health. While it is widely acknowledged that there is no known ideal or maximum number or mix of CHW job tasks that will ensure the highest level of CHW productivity, the WHO suggests that success is more likely when CHWs have a clear job description, a limited number of tasks, standardized protocols, and job aids that align with their training [83,86]. These suggestions support participant feedback advocating for a decreased workload through increasing the number of CHWs and offering skills training opportunities. It is crucial to consider various stakeholders engagement need because, it is difficult to achieve better health outcomes without coordinated engagement of actors beyond the health professionals and health sector [27,87].

Furthermore, our finding highlights that the Ethiopian health polices/ fundings are under the influence of multi-stakeholder/donors. Despite the increased efforts of actors in the Ethiopian health system overtime, the existing engagements are project based and short-term result oriented, posing a challenge to build a sustainability health system. Donor-driven approach is becoming problematic and falling to ensure a resilient health system due to some of the reasons like problems of global leadership; divergent interests; problems of accountability; problems of power relation [84,88]. Also, incompatibles interest, individual actors focus on their own set of goals and priorities [89]. According to the 2005 Paris Declaration on Aid Effectiveness, aid works best when recipient governments develop ‘ownership’ of policies and programs, and donors provide long‐term financial and technical assistance [90].

Beyond the donor initiatives approach, health diplomacy should be the focus of the health policy planners and decision makers to proactively demonstrate the level of the MNCH/ community health system challenge and advocate for the available policy options to address the health system challenge. We argued that for comparable MNCH response, there is a limited proactive health diplomacy policy and practice to mobilize the international and local actors to address the major health system challenge. Health diplomacy requires a delicate combination of technical expertise, legal knowledge, and diplomatic skills that have not been systematically cultivated among either foreign service or global health professionals [91].

## Conclusion

### Policy implications of findings

Our study demonstrated the need to move beyond the current use of multi-stakeholder engagement as a buzzword in health policies to practice it through building on multi-stakeholder engagement and embedding a meaningful coproduction framework as one of the building blocks to transform Ethiopian community health systems. We provide the following recommendations:

#### Building on the Multi-stakeholder engagement.

Increasing attention is being paid to multi-stakeholder engagement to drive the development practice and has become a hot button for policymakers and healthcare leaders. Our findings have indicated that stakeholder engagement is an important vehicle for mobilizing and sharing knowledge, technologies, and resources to support the achievement of the SDGs through promoting effective public and non-government actors partnerships and building on the experience and resourcing strategies of partnerships [92]. Our findings support the idea that individuals or groups involved in or affected by health- and healthcare-related polices should have a meaningful role in the planning, practice, and evaluation [44,93]. Mitigating the multi-stakeholders’ challenge requires multi-sector alignment (relational coupling), operational perception alignment (cognitive coupling) and goal and strategic alignment (material coupling) [94]. Study implications show the promising efforts of Ethiopia’s aspects, such as an embedded integrated community health system, promising macro-level multi-stakeholder and community-level engagement and coproduction aspects. Therefore, we recommend that efforts be guided by a multi-stakeholder engagement strategy to coordinate the process, monitor, and evaluate the impact in the coproduction framework. Vertical and horizontal alignment, continuum of and sustainable engagement practice, and systemic coordination platforms should be basic component of effective multi-stakeholders’ engagement.

#### Embedding a meaningful coproduction framework.

Our findings indicate that embedding a meaningful and continuous coproduction framework is crucial to address the multi-stakeholders’ challenge to optimize the MNCH outcomes and a resilient community health system in rural Ethiopia. We recommend adhering to a co-production framework that places citizens at the centre of public service design and production [29] and provides a space for relationship building, knowledge sharing and capacity building of all partners involved. Co-production asserts proactive consented stakeholder engagement in all levels of project cycle defining the problem, designing, and delivering the solution, and evaluating the outcome, either with professionals or independently [95,96]. We recommend co-production with potential stakeholders, including end users has been achieving promising results in developing and adapting global health interventions, the involvement of potential end users in the design, delivery and evaluation of interventions [30]. Our study highlights the importance of coproduction, stresses on the power of co-creation to address critical gaps including poor coordination of government and non-governmental actors to enhance the community level health system [97]. Realizing effective coproduction framework can be achieved by “complementarity‟ and “embeddedness‟ [28], inclusive and meaningful multi-stakeholder joint prioritization, planning, mobilization, implementation, decision-making and evaluation platform is fundamental. Coproduction demands shift in the balance of power, building a shared understanding between stakeholders including service users, researchers, policy makers, practitioners, and managers [98]. Coproduction is increasingly recognized as a critical strategy for addressing health system challenges in LMICs by fostering inclusive, context-specific solutions. A 2024 systematic review identified over 20 public health interventions across LMICs that successfully used coproduction or co-creation approaches, emphasizing the importance of local stakeholder involvement and contextual tailoring [99]. For example, participatory planning in Uganda and India improved hygiene and maternal health outcomes, respectively, by aligning interventions with community needs. These models demonstrate that coproduction enhances trust, relevance, and sustainability, key to overcoming systemic barriers in under-resourced health systems [100].

To conclude, our study reveals mixed policy and practice-related results, the current multi-stakeholder engagement is necessary but insufficient and fragmented to coproduce optimized MNCH outcomes and UHC. In the context of multi-stakeholder approach, our analysis revealed the multilevel barriers to and facilitators of coproduction in the community health landscape in rural Ethiopia. Given the recurring socioeconomic challenges including fragile health system and low level of health outcomes, the Ethiopian government alone cannot address all the challenges in short time or years. Beyond the existing fragmented multi-stakeholder engagement, a new way of thinking is required to effectively optimize existing and emerging multi-stakeholder engagement towards the coproduction of optimized MNCH and a resilient community health system in Ethiopia. A coproduction framework leverages the alignment, intersection, and mutual reinforcement of multi-stakeholder synergy throughout the CHWs program cycle through shared power and joint assessment, planning, implementing, decision making and evaluating. Fostering effective multi-stakeholder engagement synergy requires balanced shared power, alignment to community priority, systemic mapping, coordination and monitoring, and continuum and sustainability of engagement strategy.

Beyond donor initiative approaches, proactive health diplomacy strategy is also important to sustain the existing and attract new actors to realize sustainable positive health outcomes and a resilient community health policy and strategy. Our analysis shows that there is limited engagement of Academia/ research institute and private sector in Ethiopian community health systems. Hence, more evidence is required on how the muti-stakeholders synergy should be geared towards coproduction of sustainable development including resilient health outcome in Ethiopia and beyond.

## Supporting information

S1 File Consolidated COREQ. (DOCX)

S2 File List of Ethiopian HEP and related MNCH policy. (DOCX)

S3 File Supplementary -The Ethiopian Public Health Institute REB. (PDF)

S4 File Supplementary The University of Ottawa Research Ethics- REB Approval. (PDF)

S5 File Supplementary-The sociodemographic characteristics of FGDs participants. (DOCX)

S6 File Supplementary - Additional quotes, result section. (DOCX)

## Abbreviations

CE: Community Engagement
CHW: Community Health Work
CHWs: Community health workers
CSO: Civil -Society Organization
FGD: Focus Group Discussion
FMOH/MoH: Federal Ministry of Health
HEWs: Health Extension Workers
HSP: Health Sector Development Program
HP: Health Post
HSTP: Health Sector Transformation Plan
KII: Key informant In-depth Interview
LMICs: Low- and Middle-Income Countries
MDG: Millennium Development Goal
MDGs: Millennium development goals
MMR: Maternal Mortality Rate
MNCH: Maternal Newborn and Child Health
NGOs: Non-Governmental Organization
SDGs: Sustainable Development Goals
U5MR: Under Five Mortality Rate
UN: United Nations
WHO: World Health Organization.
