# Supplementary material for: Transforming multi-stakeholder engagement towards coproduction of optimized maternal, newborn, and child health and a resilient community health system in rural Ethiopia: A qualitative study
Source: PLoS One. 2025 Aug 26;20(8):e0330159. doi: 10.1371/journal.pone.0330159 (PMC12380333; doi:10.1371/journal.pone.0330159)
Supplement: S1 File — (DOCX) [file pone.0330159.s001.docx]

Title of the study: Beyond the buzzword: Transforming the multi-stakeholder engagement towards coproduction of optimized Maternal, Newborn, and Child Health and a resilient community health system in rural Ethiopia: Qualitative Case study

Consolidated criteria for reporting qualitative studies (COREQ): 32-item checklist:

| **No. Item** | **Guide questions/description** | **Reported on Page #** |
| --- | --- | --- |
| **Domain 1: Research team and reﬂexivity** |  |  |
| *Personal Characteristics* |  |  |
| 1. Interviewer/facilitator | **Which author/s conducted the interview or focus group?**  AG coordinated and directed interviews | Methods, page 8 |
| 2. Credentials | **What were the researcher’s credentials? E.g. Ph.D., MD**  The authors’ credentials are as follows:   - Akalewold T. geberemeskel, MSc, PhD-c - Ogochukwu Udenigwe, MSc, PhD-c - Josephine Etowa, MSc, PhD - Sanni Yaya, MSc,PhD | N/A |
| 3. Occupation | **What was their occupation at the time of the study?**  AG: Doctoral Candidate  OU: Doctoral Candidate  SY: Supervisor, Professor | N/A |
| 4. Gender | **Was the researcher male or female?**  The authors’ identified genders are as follows:  AG: Male  OU: Female  SY: Male | N/A |
| 5. Experience and training | **What experience or training did the researcher have?**  -AG: quantitative and qualitative training and extensive experience in maternal and child health, including sexual and reproductive health program intervention coordination, monitoring and Evaluation,  - OU: quantitative and qualitative research training and experience in qualitative research, and extensive experience in maternal and child health,  - SY: quantitative and qualitative training and extensive experience in global maternal and child health, including sexual and reproductive health care. | N/A |
| *Relationship with participants* |  |  |
| 6. Relationship established | **Was a relationship established prior to study commencement?**  AG has more than 8 years in community health program management in Ethiopia and worked in different contexts of health programs implementation and evaluation in Ethiopia, had established communication with Mr. Abebe Tadess, who was the former west Shewa Zone Health Director. | N/A |
| 7. Participant knowledge of the interviewer | **What did the participants know about the researcher? e.g. personal goals, reasons for doing the research.**  The study objectives were disclosed to participants as part of the informed consent procedure. | Methods, page 10-12 |
| 8. Interviewer characteristics | **What characteristics were reported about the interviewer/facilitator? e.g. Bias, assumptions, reasons and interests in the research topic**  A research assistant was assigned to take notes during the FGDs after a brief training about the research ethics and process by AG. The research assistant was fluent in local Oromo language(FGD participants’ working language), has a BSc degree and related work experience. | Methods, page 11 |
| **Domain 2: study design** |  |  |
| *Theoretical framework* |  |  |
| 9. Methodological orientation and Theory | **What methodological orientation was stated to underpin the study? e.g. grounded theory, discourse analysis, ethnography, phenomenology, content analysis**  This study is a qualitative case study using document review, Focus group Discussion (FGDs)) and Key Informant Interview (KII). Qualitative case study is a research methodology that helps in the exploration of a phenomenon within some particular context. Desai and Potter (48) stressed that development study requires the use of a wide range of research methods: the mix of methods enables the different techniques and their results to be compared against each other, allowing judgements to be made as to which method (or combination of methods) is the most appropriate for any particular purpose. In a case study, a real time phenomenon is explored within its naturally occurring context, with the aim of answering the “how” and “why” questions (49,50).  Our study was guided by different theoretical frameworks to inform the research processes and analysis. First, the socio-ecological framework to inform the description of the multilevel determinants of the CHWs’ program effectiveness(44,45). Second, our study was informed by the WHO’s Health System Framework, a leading structure of discourse on health systems building(45). Third, we used the Synergetic model: synergetic model shifts the focus from state-private business coproduction to ideas that involve state-society organization cooperation (28). Forth, we draw on Kingdon’s multiple streams framework(47).: Kingdon’s approach provides the conceptual framework for the analysis of the three streams – problems, policies, and politics.  A purposive sampling technique was used to recruit the FGD and KII participants. | Methods, page |
| *Participant selection* |  |  |
| 10. Sampling | **How were participants selected? e.g. purposive, convenience, consecutive, snowball**  AG identified and purposefully recruited all participants, CHWs/HEWs had to be adults ranging from the ages of 21 and 64 who had been residents of the district (work site), in West Shewa Zone health office. The participants must have had one-year experience working as a CHWs/MNCH before COVID-19; three and more years of CHW/MNCH work experience in a rural area; over one year of HEWs national training, must be full time employee and be salaried. The study did not exclude HEWs/CHWs by their gender or sex; based on the lead author’s practical experience, it was expected that CHWs would be all Female in agrarian areas of Ethiopia.  **KII participant:** Key informants were recruited based on their known involvement in the policy process leading to the planning and the implementation of CHWs/ MNCH program. Snowball sampling were used to recruit participants, suggested by other participants | Methods, page 8-10 |
| 11. Method of approach | **How were participants approached? e.g. face-to-face, telephone, mail, email**  **FGD recruitment:** Using purposive sampling, AG worked with the Oromia Regional Health Bureau (ORHB) and zone health administration in an attempt to select the two districts. Then AG contacted and worked with the district health office in an attempt to recruit potential FGD participants to send the recruitment poster to potential participants via the districts’ regular means of communication; telegram, email and announcements during review meeting.  **KII recruitment:** Snowball sampling was used to recruit participants. AG worked with the MoH, ORHB, and zone health administration in an attempt to select the first potential participant in the KII. The PI started by sending an invitation email with the study, KII recruitment poster. Then the chain continued with only one referral from potential participants.  We used a first come-first served basis for enrolment. Prior to participating in this study, participants were asked to provide their free and informed consent by signing a consent form  All FGDs and 11 KII were conducted face-to-face, 1 KII was conducted using zoom. | Methods, page 8-10 |
| 12. Sample size | **How many participants were in the study?**  A total of 16 female CHWs in the FGDs.  A total of 12(8 males & 4 Females) policymakers participated in the study. | Methods, page 12 |
| 13. Non-participation | **How many people refused to participate or dropped out? Reasons**?  None | N/A |
| *Setting* |  |  |
| 14. Setting of data collection | **Where was the data collected? e.g. home, clinic, workplace**  Data collection took place at different locations depending on participants. The FGDs were conducted in a rented hotel hall while KII with policy makers were conducted in convenient locations for participants such as their offices. | Methods, Page 10 |
| 15. Presence of non-participants | **Was anyone else present besides the participants and researchers?**  No, non-participants were present during the group discussions. | N/A |
| 16. Description of sample | **What are the important characteristics of the sample? e.g. demographic data**  In FGD, a total of 16 Female health extension from tow district of west Shewa Zone. All the FGD participants, HEWs/CHWs are female, and majority 15(93.75 %) of them are in the age range of 25-40 and only one person is in the age range of 20-24 years old. In terms of their current relationship or marital status, married 12(75%), single 2(12.5%) and widowed / separated/divorced 2(12.5%). The highest level of education or training that they have completed: collage diploma 12(75%) and over one year HEWs training 4(25%).  KII: A total of twelve public health experts were recruited to participate in the KIIs. Eight participants (66.6%) were recruited from three levels of government health structures (three participants from MoH, two participants from Oromiya Health Bureau, and three participants from West Shewa and two district level health offices). Four (33.3%) participants were recruited from NGOs (two from the National level and two from the regional level. In terms of gender, four (33.3%) women and eight men (66.6%) participated. Most of the participants have post-graduate level (MSc) educational backgrounds in health and related study programs and they have more than 10 years of public health related extensive experience in different levels of responsibilities. | Page#12 |
| *Data collection* |  |  |
| 17. Interview guide | **Were questions, prompts, guides provided by the authors? Was it pilot tested?**  AG carefully crafted FGD and KII guides in English and translated to Oromo and Amharic languages. The guides were carefully crafted following the theoretical frameworks.  The KII was guide was piloted with two health policy experts, in West Shewa Zone, Oromiya region and they were not participated/contacted during the actual data collection. Based on their response minor modificataion was applied on the KII guide for the final version of the gude. | Methods, page 9-10 |
| 18. Repeat interviews | **Were repeat inter views carried out? If yes, how many?**  Repeat interviews were not carried out. | N/A |
| 19. Audio/visual recording | **Did the research use audio or visual recording to collect the data?**  FGD and KII were audio-recorded after obtaining participants’ permission to record. | Methods, page 10 |
| 20. Field notes | **Were ﬁeld notes made during and/or after the interview or focus group?**  Yes, filed note was aken by trained research assistants during FGD | Methods, page 9 |
| 21. Duration | **What was the duration of the inter views or focus group?**  FGD lasted about 80-90 minutes and ended when no further issues arose. Each KII lasted approximately 60-90 minutes. | Methods, page 9 |
| 22. Data saturation | **Was data saturation discussed?**  The principal investigator (AG) estimated the saturation of data between 6-8 individuals based on previous similar approaches.  Yin (34) suggests multiple cases enable replication logic, allowing researchers to confirm or disconfirm inferences drawn from each case. We conducted two FGDs , the move from single-case to paired comparison offers a balanced combination of descriptive depth and analytical challenge that progressively declines as more cases are added.  The principal investigator (AG) estimated the saturation of data between 10-12 interviews.  For KII, data saturation can be attained in as little as 12 interviews depending on the diversity of data and the sample population, however, the concept of data saturation is also contested within research designs such as qualitative description that stress the uniqueness of each individual’s experience. The authors acknowledge that information obtained from 12 public health policy experets may never truly reach data saturation, the key however, was to strive to attain thick and rich data. Based on the diverse policymakers interviewed for this study, the authors believe that the data obtained is detailed, nuanced and intricate. | Methods, page 10 |
| 23. Transcripts returned | **Were transcripts returned to participants for comment and/or correction?**  No transcript was returned to participant | N/A |
| **Domain 3: analysis and ﬁndings** |  |  |
| *Data analysis* |  |  |
| 24. Number of data coders | **How many data coders coded the data?**  The authors AG, OU,JE and SY coded the data | Methods, page 9-10 |
| 25. Description of the coding tree | **Did authors provide a description of the coding tree?**  The transcript was read and coded based on identified similarities and patterns in the data. | Methods, page 10 |
| 26. Derivation of themes | **Were themes identiﬁed in advance or derived from the data?**  Data analysis followed the analytical strategies for qualitative description. Codes were developed after an initial review of the transcripts. First, after familiarization to the transcript , AG & OU read the data line by line, recorded insights, and proceeded to coded the transcript , FGD and KII, they coded individually and compared their codes. After debriefing and consensus the transcript was coded accordingly. After debriefing, JE, SY and OU, randomly chosen and compared to check alignment or discrepancies of the coding of the transcript. Next, coded information were sorted to identify patterns and themes from which similarities and differences were identified and extracted for further consideration and analysis. Similar themes generated sub-categories which gave a more general description of the content. Emerged themes were based on an iterative process of inductive and deductive approaches. | Methods, page 9 |
| 27. Software | **What software, if applicable, was used to manage the data?**  No software was used | N/A |
| 28. Participant checking | **Did participants provide feedback on the ﬁndings?**  No, the participants did not provide feedback on the findings | N/A |
| *Reporting* |  |  |
| 29. Quotations presented | **Were participant quotations presented to illustrate the themes/ﬁndings? Was each quotation identiﬁed? e.g. participant number**  Participants’ quotations were presented to illustrate themes and findings. | Results, pages 13-20, 37 |
| 30. Data and ﬁndings consistent | **Was there consistency between the data presented and the ﬁndings?**  Yes. | Results, pages 13-24 |
| 31. Clarity of major themes | **Were major themes clearly presented in the ﬁndings?**  Yes, we organized the findings by major themes. | Results, pages 21-24 |
| 32. Clarity of minor themes | **Is there a description of diverse cases or discussion of minor themes?**  Yes, we discussed minor themes in the manuscript and situated them within the broader literature. | Discussion, pages 13-24 |
