# Supplementary material for: Transforming multi-stakeholder engagement towards coproduction of optimized maternal, newborn, and child health and a resilient community health system in rural Ethiopia: A qualitative study
Source: PLoS One. 2025 Aug 26;20(8):e0330159. doi: 10.1371/journal.pone.0330159 (PMC12380333; doi:10.1371/journal.pone.0330159)
Supplement: S2 File — (DOCX) [file pone.0330159.s002.docx]

**S-2 ­- List of policy documents**

List of Ethiopian HEP and related MNCH policy documents

| # | Title of the document |
| --- | --- |
| 1 | MoH.2020. Realizing universal health coverage through primary healthcare A Roadmap for Optimizing the Ethiopian Health Extension Program 2020 – 2035 <http://repository.iifphc.org/handle/123456789/1175> |
| 2 | MoH. 2020. An implementation manual for Optimizing Health Extension Program  Ministry of Health, Addis Ababa, Ethiopia |
| 3 | Roadmap towards maximizing newborn and child survival and wellbeing by 2030 ministry of health, Ethiopia January 2022 |
| 4 | MoH. 2020. Integrated management of newborn and childhood illness facilitator guide Addis Ababa, Ethiopia |
| 5 | MoH. 2021. National strategy for newborn and child health and development in Ethiopia November 2021 |
| 6 | MoH. 2021. Integrated management of newborn and childhood illness May 2021 Addis Ababa, Ethiopia |
| 7 | MoH.2021. Ethiopia National Expanded Program on Immunization comprehensive multi-year plan (2021-2025) Federal Ministry of Health Addis Ababa July 2021 |
| 8 | MoH.2021. Ethiopia National Expanded Program on Immunization comprehensive multi-year plan (2021-2025) Federal Ministry of Health Addis Ababa July 2021 |
| 9 | MoH.2021. Family planning service integration national implementation GUIDELINE MCHN directorate, MoH August, 2021 Addis Ababa, Ethiopia |
| 10 | MoH.2022. Health Equity and System Strengthening Monitoring and Evaluation Guideline June, 2022 Addis Ababa, Ethiopia |
| 11 | MoH.2022. National Antenatal Care Guideline, Ensuring Positive Pregnancy Experience! February 2022 |
| 12 | MoH.2013. National strategic plan for elimination of obstetric fistula 2021-2025 (2013-2017 EFY) |
| 13 | MoH.2021. Reproductive health (RH)-StrategicPlan_2021 |
| 14 | MoH.2021. Federal Democratic Republic of Ethiopia National Food and Nutrition Strategy May 2021 |
| 15 | A Roadmap for Optimizing the Ethiopian Health Extension Program 2020 - 2035 1 st Edition July 2020 Addis Ababa, Ethiopia |
| 16 | ORHB. 2022. Biiroo Fayyaa Oromiyaa Daayirektoretii Wal’aansa Fi Hooggansa Dhaabbile Fayyaa Karoora Hojii Garee Tajaajila Sagantaa Ekisteenshini Fayyaa Kan Bara 2015 Hagaya 2014 Finfinnee |
| 17 | MoH.2020. Revised Community Engagement Approaches in Rural Agrarian Ethiopia: An Implementation Guide for piloting of the community engagement approaches, August 2020, Addis Ababa |
| 18 | MoH.2022. የጤና ኤክስቴንሽን ፕሮግራምና የመጀመሪያ ደረጃ ጤና ክብካቤ ዳይሬክቶሬት የ2014 በጀት ዓመት የአመታዊ ዕቅድ አፈጻጸም ሪፖርት. ቅጽ. 4. ሪፖርቱ የሚሸፍነው ጊዜ፡ ከሐምሌ 1/2013 ዓ.ም. እስከ ሰኔ 30/2014 ዓ.ም. |
| 19 | MOH _ National Implementation Guideline for Expanded Program on Immunization (Revised edition) June 2021 Addis Ababa, Ethiopia  <http://repository.iifphc.org/bitstream/handle/123456789/1681/National-Implementation-Guidline-for-Expanded-Program-on-Immunization.pdf?sequence=1&isAllowed=y> |
| 20 | Oromiya Bureau of Finance and Economic Development 2010. NGOs Affairs Work Process, revised. Terms of Collaboration Between Oromiya National Regional Government and NGOs Operating in the State, Finfinne  May 2010 |
