# Supplementary material for: Transforming multi-stakeholder engagement towards coproduction of optimized maternal, newborn, and child health and a resilient community health system in rural Ethiopia: A qualitative study
Source: PLoS One. 2025 Aug 26;20(8):e0330159. doi: 10.1371/journal.pone.0330159 (PMC12380333; doi:10.1371/journal.pone.0330159)
Supplement: S3 File — (PDF) [file pone.0330159.s003.pdf]

የኢትዮጵያ የሕብረተሰብ ጤና ኢንስቲትዩት
Ethiopian Public Health Institute
አዲስ አበባ-ኢትዮጵያ Addis Ababa, Ethiopia

ስልክ -Tel: +251 11 2133499, +251 11 2751522, ፋክስ Fax: +251 11 2758634,
የመ.ሳ.ቁ-P. O. BOX: 1242/5654 e-mail: ephi@ethionet.et
www.ephi.gov.et

ቁጥር E.P.H.I
Ref. No.
ቀን 08 SEP 2022
Date

Ethiopian Public Health Institute
Institutional Review Board (EPHI-IRB)
Certificate of Approval

Protocol number: **EPHI-IRB-462-2022**
Minutes No; 109

Protocol Title: Paths to transform community health workers program to enhance maternal, newborn and child health equity and health system resilience in rural Ethiopia	
Investigators:	Mr. Akalewold Tadesse Gebremeskel
Institute:	University of Ottawa
Study site/s	Ethiopia
Elements Reviewed (EPHI-IRB AF 01-008/02.0):	☐ Attached ✔ Not attached
Mode of Review	✔ Expedited ☐ Full Board
Decision of the meeting	✔ Approved

I. Elements approved:
1. Protocol Version No: 02
2. Protocol Version Date: 01-09-2022
3. ICF Version No.; 02
4. ICF Version Date: 01-09-2022

II. . Obligations of the PI:
1. Should comply with the standard international &national scientific and ethical guidelines
2. All amendments and changes made in protocol and consent form needs IRB approval
3. The PI should report SAE within 48 hours of the event
4. This approval certificate is valid for only one year (specified below). The PI should Submit continuation request before expire date of approval, if projects is to continue
5. Final report/Thesis and Manuscripts should be submitted to the IRB secretariat after completion of the study.

Institutional Review Board Approval Date: September 1, 2022
Approval Period: From September 01, 2021 to August 31, 2023
Follow up report expected in:
6 months ✔ 9 months ______ one year ______

EPHI-IRB Chairperson
Name Atkure Defar
Signature [signature]
Date: Sep 6

EPHI Director General
Name [signature]
Signature
Date

Mesay Hailu (PhD)
Director General

Scanned by CamScanner
