# Supplementary material for: Transforming multi-stakeholder engagement towards coproduction of optimized maternal, newborn, and child health and a resilient community health system in rural Ethiopia: A qualitative study
Source: PLoS One. 2025 Aug 26;20(8):e0330159. doi: 10.1371/journal.pone.0330159 (PMC12380333; doi:10.1371/journal.pone.0330159)
Supplement: S5 File — (DOCX) [file pone.0330159.s005.docx]

**S-3- Supplementary: The sociodemographic characteristics of FGDs participants, HEWs/CHWs**

Sociodemographic characteristics of FGD participant, HEWs/CHWs

|  | Adea Bega | % | Ejere | % | Total (Adea Berga and Ejere),% |
| --- | --- | --- | --- | --- | --- |
| 1.What is the average distance between your work site and field work/outreach site in kilometers? | | | | | |
| A. Less than 3 km | 2 | 25 | 0 | 0 | 2(12.5) |
| B. 3km to 5 km | 0 | 0 | 5 | 62.5 | 5(31.25) |
| C. Above 5km | 6 | 75 | 3 | 37.5 | 9(56.25) |
| 2. What is the most commonly used kind/ mode of transportation during field work/ outreach activities? | | | | | |
| Walking / on foot | 8 | 100 | 8 | 100 | 16(100) |
| 3. What are the most commonly used forms/ means of communication with your client (phone, home visits…)? | | | | | |
| Home to home visit | 8 | 100 | 8 | 100 | 16(100) |
| 4.What is the approximate house hold in your catchment? | | | | | |
| 300-500 | 1 | 12.5 | 0 |  | 1(6.25) |
| 501 and above | 7 | 87.5 | 8 | 100 | 15(93.75) |
| 5.What is your total work experience as a HEW (in years)? | | | | | |
| A. less than 4 years | 0 |  | 0 |  |  |
| B. 4-5 years | 0 |  | 2 | 25 | 2(12.5) |
| C. 6 and above 8 | 8 | 100 | 6 | 75 | 14(87.5) |
| 6.What do you currently identify yourself as Gender? | | | | | |
| A. Man | 0 | 0 | 0 | 0 |  |
| B. Woman | 8 | 100 | 8 | 100 | 16(100) |
| C. Other (specify): | 0 | 0 | 0 | 0 |  |
| 7.What is your CURRENT relationship or marital status? | | | | | |
| A. Single | 2 | 25 | 0 | 0 | 2(12.5) |
| B. Married | 6 | 75 | 6 | 75 | 12(75) |
| D. Widowed / Separated/Divorced | 0 | 0 | 2 | 25 | 2(12.25) |
| 8. What is the highest level of education or training that you have completed? | | | | | |
| A. Completed high school and over 1 year HEWs training | 0 | 0 | 4 | 50 | 4(25) |
| B. Completed collage diploma and over 1 year HEWs training | 8 | 100 | 4 | 50 | 12(75) |
| C. Other (specify): | 0 | 0 | 0 | 0 |  |
| 9.During the past 12 months, what was your estimated monthly personal income from all sources in Ethiopian Birr? | | | | | |
| A. 3000 and below ETB per month | 0 | 0 | 1 | 12.5 | 1(6.25) |
| B. 3,001- 5000 ETB per month | 4 | 50 | 2 | 25 | 6(37.5) |
| C. 5001 and above ETB per month | 4 | 50 | 5 | 62.5 | 9(56.25) |
| D. Prefer not to answer | 0 | 0 | 0 | 0 |  |
| 10. How old are you now? | | | | | |
| A.20-24 years old | 0 | 0 | 1 | 12.5 | 1(6.25) |
| B. 25-40 years old | 8 | 100 | 7 | 87.5 | 15(93.75) |
| C. 41 years and older | 0 | 0 | 0 | 0 |  |
