# Supplementary material for: Transforming multi-stakeholder engagement towards coproduction of optimized maternal, newborn, and child health and a resilient community health system in rural Ethiopia: A qualitative study
Source: PLoS One. 2025 Aug 26;20(8):e0330159. doi: 10.1371/journal.pone.0330159 (PMC12380333; doi:10.1371/journal.pone.0330159)
Supplement: S6 File — (DOCX) [file pone.0330159.s006.docx]

**S-6- Supplementary: Additional quotes, result section**

Additional description and quotes: In the context of multi-stakeholder approach, our analysis reviled the multilevel barriers to and facilitators of coproduction in the community health landscape in rural Ethiopia.

| Major Themes | Sub-themes | Additional: Description and quotes |
| --- | --- | --- |
| Barriers to Multi stakeholders’ engagement | Lack of vertical and horizontal alignment | The existing support approach is not well aligned to the holistic community health program system strengthening, instead it is vertically aligned to specific disease or project based on donors need.  *“We work with them during planning too and we also clearly inform them on how they can support the plan and the role they would play. However, many partner organizations are involved in software activities such as capacity building, evidence generation, training, education, etc. There is no partner organization that has pushed that much on infrastructure. When the ministry was working on health sector transformation, the participation of partners was not easy.” (KII 8, MoH, National HEWs/CHWs expert)* |
|  | Lack of continuum of and sustainable engagement practice | *“The community engagements are project based and donor- initiated … It is based on some incentive, when there is incentive there is better engagement, when the support/ project stop it also stops. … also it is a short outcome oriented, if you are doing to provide TB service and your focus would be on the result for the project year, let’s say for five-year plan. You struggle to achieve the result you should achieve in a five year, not about the sustainability. Then the focus would be on improving the demand creation to get the immediate result. This is a kind of marketing or selling your product not for long term impact. These are a challenge for sustainability.” (KII 12 Regional NGO, Public health Expert)* |
|  | Lack of systemic coordination platforms | *More quotes could be moved from the main text here* |
| Facilitators of multi stakeholders’ engagement | Embedded integrated community health system | CHWs program is very relevant program for the health of mothers and children in a disadvantaged context to access basic health service at their neighborhood.  *“I believe that there is no substitute for the health of our citizens living in rural areas, especially when it comes to the health of mothers and children. Because the first health extension works in their community and village (Kebele). Second, the health extension experts are women and they easily understand the problems of mothers and children.” (KII 8, MoH, National HEWs/CHWs expert).*  *“One of our benefits is getting the chance to serve our own community at their neighborhood. The community were used to travel long distance to vaccinate their children’s; also, mothers need to travel long for ANC. In this case due to the challenge there were high dropout. Now, we are contribution is this aspect, our community are benefiting from our service and improving their health.” (FGD participant, From Ejere District)*  The Ethiopian CHWs/ HEP program  *“We provide services like vaccination, home to home visit and education, growth monitoring for children of under two-year, pregnant women follow up, ANC services, we treat under five children for diarrhea.” (FGD participant, From Adea Berga District* |
|  | Promising macro-level multi-stakeholder engagement and coproduction aspects | Perspective on the macro-level multi-stakeholder engagement platform indicates the need for better accountability programs community participation.  *“With the limited government budget, it is not possible to achieve our health goal. To improve the health system including the HEWs program the participation of, donors, partners, and society is important.*  *“The HEWs program optimization requires a large investment, so it is not expected to achieve just through limited events. In this way, it is not possible for the government alone to win. Then serious consultation, advocacy, and resource mobilization is required.” (KII 7, MoH, Primary health system expert).*  *“In our experience they have a contribution, we have produced better accountability programs in the health sector to bring about change through community participation. We promote the community understand the service standard and evaluate the provision using the community scorecard.” (KII 12 Regional NGO, Public health Expert)*  *“NGOs and government should work together; they have the same target community and community service. When there is no coordination and no unity the level of addressing a problem would also drop.”* ***(****KII 5, Regional health bureau, Primary health expert)*  There is a macro level promising initiatives of joint planning, implementation and evaluation initiatives and joint technical working group and a steering committee in every level.  *“As a ministry of health, we involve all levels from woreda to the ministry of health on one plan, one budget, and one report. This is one part of the efforts of the MoH to engage the grass root goes down to the district and coordinate.” (KII 8, MoH, National HEWs/CHWs expert).*  *“Most of the health budgets come from partners, partners were covering the budget for capacity-building activities, and partners also had a significant role in terms of supply and logistics.” (KII 9, Zonal level health MNCH expert).*  *“Yes, various donors help the health sector program. The health sector is one of the beneficiaries of SDGs resource pooled from different donors. So, health sector uses more doners budget. So, as you said there should be a way to enhance the domestic resource mobilization. Increasing the export item to improve the economy of the country. I don’t think that there is a simple way to’ move out of aid dependency in a short time.” (KII 8, MoH, National HEWs/CHWs expert).*  Majority of CHWs health post equipment’s are donated by UNICEF.  *“The government is not purchasing any equipment for HEWs., most of the equipment’s in the health post are donated by UNICEF. So, this way it is possible to strengthen the health system. “(KII 4, District health office, HEP experts)*  *“Currently UNICEF is working with us, helping the health sector. In our current situation, most of the work is being covered by the support form partner organizations. They are supporting the government in terms of supply, medicine, budget and others.” (KII 9, Zonal level health MNCH expert).*  *“I think as a government worker, joint planning, joint implementation and joint evaluation with partner organizations is crucial. If the work plan of the partner is not explicitly clear it is difficult to implement and address the intended target community, Mothers, and children in this case.” (KII 9, Zonal level health MNCH expert).*  There are different platforms of community engagement in Ethiopia. These include indigenous institutions like ‘Idir’ ‘Ikub’ and Traditional Birth Attendants and CHWs program-based groups like Women development groups.  *“There are multiple names, they say community engagement, community dialogue, community conversation, some times they say community participation, some times they say one to five, there is a political attachment/ force behind.” (KII 12 Regional NGO, Public health Expert)*  *“There are strong social structures in our community, like ‘Iqub’ ‘idir’ to utilize their potential beyond burial service in health service improvement. Religious institutions are also considered. There is a direction to fully engage the social structures”. (KII 7, MoH, Primary health system expert).*  Beyond as a service user, the grass root community has been playing considerable roles as a key stakeholder, Aspects of community engagement includes demand creator and health promoters, and potential source of resource mobilization.  *“It has a great importance. We attend the community gathering, then we address our message on mothers and children health and family planning, which is part of community participation. So, the community can disseminate the information to their respective village, they can mobilize for community during vaccination and collect the children at specific site, they can collect and crops for women during delivery.“FGD participant, Ejere District)*  *“…they help us, they mobilize the community to provide monthly vaccination for children of underage one and deworming for children. As related to Covid, our community are playing various role to ensure handwashing every time, maintaining clear living environment, constructing public toilet along the road side of their respective village with an intention to prevent the community's exposure to diseases including COVID-19.” (FGD participant, From Adea Berga District)*  *” Community engagement should start from effective communication with the community. To realize community engagement, from the beginning mapping the stakeholder like influentials in the project site is important. If all the stakeholders engage on identifying: What is the gap or the challenge? What is the existing potential in the community? How the community need that problem it to be addressed or what is the solution? Then, if you already have planned together, you would not complain while eating, dinking and you will enjoy every thing together. If you jointly design the cloth you wear, you will accept and use it.” (KII 12 Regional NGO, Public health Expert)*  “*…the community can do whatever it can as long as there is transparent discussion/ communication on how to improve and contribute to the health system. I can say that there is no problem in the community. Currently, there is no specific project implemented by government alone, with out community participation, the government is mostly emphasizing Health and human resource, even if it is not enough.” (KII 4, District health office, HEP experts)*  For effective community engagement, “It is important to talk with the community on ‘the how part’”.  *It is important to talk with the community on” the how part”. If you acknowledge the value and the culture of the community and able to jointly plan the community is willing to support and own it. But, if it is for demand creation, is just for selling your products ……if you choose the design the style, the colour and the like there is no reason for the community not to own the project.* |
|  | Community-level engagement and coproduction aspects | *“The biggest thing to strengthen the health system is finance. That is, if it needs to be strengthened. Our health system, especially, most of its financing comes from abroad, and since the financing coming from abroad is decreasing in the actual situation, one of the things that should be done is domestic financing. There is a need to strengthen CBHI and Social Health Insurance because there are things that have been started as health insurance. That is one thing, and in addition, this will help to provide and strengthen sustainable health services in the future. It means that you need to get out of dependency quickly.” (KII 7, MoH, Primary health system expert)* |
