# Supplementary material for: Transforming multi-stakeholder engagement towards coproduction of optimized maternal, newborn, and child health and a resilient community health system in rural Ethiopia: A qualitative study
Source: PLoS One. 2025 Aug 26;20(8):e0330159. doi: 10.1371/journal.pone.0330159 (PMC12380333; doi:10.1371/journal.pone.0330159)
Supplement: Attachment — Submitted filename: Response to Reviewers.docx [file pone.0330159.s007.docx]

'Response to Reviewers- PONE-D-23-23186

Transforming multi-stakeholder engagement towards coproduction of optimized maternal, newborn, and child health and a resilient community health system in rural Ethiopia: A qualitative study

Thank you Dr. Rachidatou Compaoré for reviewing and positive feedback on our manuscript.

|  | Review Comments | Response |
| --- | --- | --- |
|  | If you would like to make changes to your financial disclosure, please include your updated statement in your cover letter. Guidelines for resubmitting your figure files are available below the reviewer comments at the end of this letter. | Thank you. There is no change on the financial disclosure. |
| Additional Editor Comments: | In the abstract, specify the period of study, report the results so that they are more specific to what you found in your study, and ensure that the recommendations made are based on the reported findings. | Thank you for the suggestion. See page #2 “Data collection period was between September 15, 2022, to end of November28, 2022.” |
|  | In the introduction, check the accuracy of the reported data. | Thank you for the suggestion. We have revised the citation and provided the original reference.  “Primary health care systems (PRIMASYS): case study from Ethiopia, abridged version. Geneva: World Health Organization; 2017. Licence: CC BY-NC-SA 3.0 IGO.” |
|  | In the results section, structure the results to give a common thread to the readers and present the different themes in a balanced way. | Thank you for the suggestion. We carefully considered your suggestion with out affecting the coherence and completeness of the result section. |
|  | Ensure that all abbreviations have been defined from the first use. | Thank you for bringing that to our attention. We have revised the abbreviation section and included the missing. |
|  | Ensure that all references meet the journal's recommendations for citations. | Thank you for the suggestion. Thank you for bringing that to our attention. We have revised the reference section accordingly. |
| Reviewers' comments: |  |  |
| **Reviewer #1:** | Thank you for the study that highlights a growing interest and need to incorporate community voices and ideas in what affects their own health in an attempt to get communities to own and become responsible for their health. the article is generally well written, with a few suggestions to consider. | Thank you for your positive feedback |
|  | Please check that the numbers are correct on Introduction, page 4, paragraph 4 on the proportion of health finance. | Thank you for the suggestion. We have revised the citation and provided the original reference.  “Primary health care systems (PRIMASYS): case study from Ethiopia, abridged version. Geneva: World Health Organization; 2017. Licence: CC BY-NC-SA 3.0 IGO.” |
|  | In the results section, I suggest that the authors reduce text on the much longer themes and have a balance in the number of quotes presented across all themes. | Thank you for your suggestion we have tried to consider this suggestion with out impacting the coherence and completeness of the result. |
|  | Under Barriers, "the challenges of alignment", "lack of systemic coordination and patforms" and inadequate coordination and implementation capacity" seem to refer to the same thing in their current presentation. | Thank you for pointing this out. We have combined them now. “Inadequate systemic coordination and implementation capacity”, see on page#15 |
|  | Abbreviation list is not complete, e.g. SSA not included. | Thank you for bringing that to our attention. We have revised the abbreviation section and included the missing. |
|  | There a few typing errors. e.g. finding vs findings | Thank you for bringing that to our attention. We have revised this as needed. |
|  | Reference: there is not consistency in the reference list, stick to one style and and be consistent to what you choose. Some years missing, use of short or full names for journal names, use of month and date for some and not for others. | Thank you for your pointing out this. Thank you for bringing that to our attention. We have revised. See page 26 |
| **Reviewer #2:** |  | Thank you for your positive feedback |
|  | In the abstract section-Methods: We conducted a qualitative case study – not recommended in research writing to use We/I – instead better - A qualitative case study was conducted … | Thank you for your suggestion and we appreciate your point. |
|  | Check and correct all – in the case of using abbreviation in the first place write the full word (abbreviation). | Thank you for your pointing out this. |
|  | In the abstract the conclusion is not supported by the result stated? | We appreciate your suggestion. We tried to realign the result section with the conclusion. |
|  | Methods In research setting – The authors wrote more detail about Ethiopia but focus on the study setting. In this research, the nested nature of the case study encompasses two distinct populations including HEWs/CHWs (16 FGD participant), and subnational and national public health policy actors (12 KII participant). The authors do not include the community or service users – so lack triangulation in this aspect. If you can better include. | Thank you for your pointing out this. Our study is based on the service providers perspective; accordingly, we have added more information about the case.  **“Case study setting:** The case study is built up on the perspective of the multilevel community health program actors including national to, regional to, zonal to and onsite service provides (CHWs). Following Yin (50) embedded multiple case study approach we collected data from different levels or sources to enhance balanced combination of descriptive depth and analytical challenge as more cases are added(52). Such comparative methods enable generalizations of findings using between cases and with in case using multiple units of analysis (51). In this embedded multiple case study, the nested nature of the case study encompasses two distinct populations including HEWs/CHWs (16 FGD participant), and subnational(regional, zonal and district) community health program implementers and national public health policy actors (12 KII participant). These populations are spread over three geographical areas with respect to the scope of their work: West Shewa Zone CHWs, Oromia Region (subnational) and Ethiopia (national) public health policy actors.” See on page #6. |
|  | KII participants Key Informants were recruited based on their known involvement in the policy process leading to the planning and the implementation of CHWs/ MNCH program. The authors need specify the number from working at regional and ministry separately – because these are responsible for policy issues. | Thank you for your pointing out this. we have clarified it on page #10.  ” A total of twelve public health experts were recruited to participate in the Key Informant Interviews (KIIs). Eight participants (66.6%) were recruited from three levels of government health structures (three participants from MoH, two participants from ORHB, and three participants from West Shewa and two district level health offices). Four (33.3%) participants were recruited from NGOs (two from the National level and two from the regional level.” |
