# Supplementary material for: Transforming multi-stakeholder engagement towards coproduction of optimized maternal, newborn, and child health and a resilient community health system in rural Ethiopia: A qualitative study
Source: PLoS One. 2025 Aug 26;20(8):e0330159. doi: 10.1371/journal.pone.0330159 (PMC12380333; doi:10.1371/journal.pone.0330159)
Supplement: Attachment — Submitted filename: Response to Reviwers-24 May.docx [file pone.0330159.s008.docx]

**Response to Reviews**

PONE-D-23-23186R1

Transforming multi-stakeholder engagement towards coproduction of optimized maternal, newborn, and child health and a resilient community health system in rural Ethiopia: A qualitative study

Dear Dr. Tatchalerm Sudhipongpracha

Thank you for your critical and valuable feedback and continuous support. We have addressed all the comments in a line-by-line manner with more detailed descriptions and information.

|  | Reviewers feedback | Authors response |
| --- | --- | --- |
|  | Reviewer #3: | The authors thank the reviewer for their feedback, which we believe has strengthened our paper. |
|  | **1. Addressing PLOS ONE’s open data policy by specifying whether anonymized transcripts or coding frameworks can be shared.** | The authors thank the reviewer for their feedback, which we believe has strengthened our paper.  The authors have updated this as follows:  Page 26: The authors acknowledge PLOS ONE’s recommendation to make all data and related metadata available through appropriate public data repositories. However, the interview data in its current form cannot be shared publicly as it contains potentially identifiable sensitive information regarding the participants and their roles. Therefore, it would be unethical to make it public and would undermine the ethical committee agreement and consent process. Anonymized data can be requested from the Research Ethics Boards (REB) at the University of Ottawa at ethics@uottawa.ca by researchers who meet the criteria for access to confidential data. All other relevant data are presented within the article. |
|  | **2. Expand the conclusion by providing more actionable recommendations on how policymakers and practitioners can implement the findings.** | Thank you for your critical suggestion. Please see on Page 24: The authors have included actionable recommendations, as suggested by the reviewer. For example, we recommended that efforts be guided by a multi-stakeholder engagement strategy to coordinate the process, monitor, and evaluate the impact in the coproduction framework. |
|  | **Reviewer #4:** | The authors thank the reviewer for their feedback, which we believe has strengthened our paper. |
|  | Introduction (Pages 1-5: Page 5**- I would like to see the figure of the final theoretical framework.** | Thank you for your valuable feedback. We have now included a figure illustrating the theoretical framework, which is based on the multiple theoretical perspectives that guided this study. Please see on Page#6 |
|  | **The section could clarify how multi-stakeholder engagement differs from traditional public-private partnerships.** | The authors have clarified that multi-stakeholder engagement encompasses various actors, such as governments, the private sector, civil society, academia, and international organizations. Unlike public-private partnerships, which mainly focus on formal collaboration between state and private entities, multistakeholder engagements tackle complex challenges that necessitate diverse expertise and resources. These engagements prioritize the active involvement and contributions of all stakeholders, including civil society and the public |
|  | **Consider briefly discussing power dynamics (government vs. NGOs, local vs. international stakeholders).** | Thank you for your valuable suggestion. The authors earlier addressed power dynamics in the discussion section, we have however added earlier on in the paper that “Power dynamics and their expression in multi-stakeholder relationships are crucial factors, as the values, intentions, and interactions of both individuals and organizations largely influence the connections between actors. This research acknowledges the potential power imbalances stemming from those who have the authority to make and influence decisions across different contexts. This topic is further examined in the discussion section of the paper.” |
|  | Methods (Pages 5-8)  **o It would be helpful to clarify why West Shewa Zone was selected.** | Thank you for your feedback. Please see on page 6, the authors have indicated that this location was chosen for research because despite the progress in other parts of the country, the West Shewa zone is still underperforming when it comes to MCH outcomes. |
|  | **You said it was translated from Amharic and Oromia languages, how did you the translation consistency and inter-rater reliability?** | Thank you for your feedback. Please see on Page 9: The authors have indicated:  A literal translation (word-for-word) was employed to maintain the original responses of participants and help readers grasp their mindset. The translation process included back translation, which involved translating the data from the source language (Oromo and Amharic languages to the target language (English) and then reverting it back to the source to resolve any ambiguities or inconsistencies. |
|  | Page 8 **- Explain why data was manually coded instead of using qualitative software like NVivo. Provide more details on how data saturation was determined and why manual coding was preferred over software.** | Thank you for your question. We acknowledge the role of softwares to support or guide the coding process e.g., the themes. The choice for manual coding was based on the manageability of the data.  The authors indicated on page 7: The principal investigator (AG) estimated the saturation of data between 10-12 interviews and 2 focus groups and 6-8 individuals based on previous similar approaches(58) |
|  | **Please add more detail on how themes were developed inductively/deductively would strengthen credibility. If it is both inductive and deductive, specify how themes emerged and how prior frameworks influenced coding.** | Thank you for your feedback. Please see on Page 9: the authors indicated that “themes were based on an iterative process of inductive and deductive approaches. Deductive approaches were drawn from existing literature and the systematic review phase and the study theoretical frameworks. In inductive approaches, themes emerged from the data and were not be forced to fit into preconceived categories. The analyses used triangulated data from multiple sources and multilevel government and non-government health policy experts and frontline CHWs, and document analysis. Given the small size of the data, we decided to code manually without employing any qualitative data analysis software |
|  | **In the recent publications by Braun & Clarke (2021), emphasized the importance of clarifying the theoretical and philosophical assumptions underlying the chosen thematic analysis procedure. Clearly state epistemological and ontological assumptions. Is the study critical realist, constructivist, or positivist? Does it assume an objective reality (realism) or multiple realities (relativism)?** | Thank you for your feedback. Please see on page 9: the authors indicate that “This study takes a fully theory-informed inductive study design (Varpio et al., 2020). In this approach, the theoretical framework informs the research questions, the approach to research, the concepts that underpin the study design, the choice of methodology, data collection, interactions with study participants, analysis processes, and the conclusions drawn. By acknowledging that reality is subjective and varies from person to person and by centering the participants as holders of knowledge, this study draws on constructivist ontology and epistemology (Braun & Clarke, 2006).” |
|  | **Results (Pages 9-15)**  **o Some quotes are long, consider summarizing or paraphrasing for clarity.** | Thank you for your feedback. The authors have adjusted long quotes and provided summaries instead. |
|  | **The concept of "donor dependency" is discussed but could be linked to alternative funding solutions.** | Thank you for this point. The authors acknowledge that while the participants mentioned alternatives to donor dependency, their responses were somewhat limited. These are included in the result section. For example,  Page 14: *“I don’t think that there is a simple way to move out of aid dependency in a short time****.*** *Yes, various donors help the health sector program. The health sector is one of the beneficiaries of SDG resources pooled from different donors. So, the health sector uses more of donors’ budget. So, as you said there should be a way to enhance domestic resource mobilization. Increasing the export item to improve the economy of the country.” (KII 8, MoH, National HEWs/CHWs expert).*  *Page 15: “I think the government should create a conducive environment. The government should engage partners as a sector. The government should recognize the sector and invite them. But the current practice is that partners with interest go to the government. It means that the initiative is from the CSOs. But should come from the government too so that different sectors can participate as one of the development actors.” (KII 12 Regional NGO, Public health Expert)*  Since we aim to produce evidence based on participants' own words, we can only present what participants said and refrain from making inferences in this section. |
|  | **Discussion (Pages 16-19)**  **o More discussion on CHW mental health/workload challenges would strengthen practical implications.** | Thank you for your feedback. Please see on page 23, the authors discussed as follows:  Furthermore, our results demonstrate that CHWs face a substantial workload due to fragmentation. Participants reported that CHWs frequently had to carry out various tasks, including health extension programs in addition to their standard duties. A systematic review that summarized CHWs’ perceived workload across low- and middle-income countries supports the observation that CHWs endure a considerable workload. This study suggests that a heavy workload can adversely affect their productivity and significantly harm both their physical and mental health. While it is widely acknowledged that there is no known ideal or maximum number or mix of CHW job tasks that will ensure the highest level of CHW productivity, the WHO suggests that success is more likely when CHWs have a clear job description, a limited number of tasks, standardized protocols, and job aids that align with their training. **These suggestions support participant feedback advocating for a decreased workload through increasing the number of CHWs and offering skills training opportunities.** |
|  | **Provide examples of successful coproduction models from other LMICs for comparison**. | Thank you for your valuable suggestion. Now we have added successful examples, please see on page 26. Coproduction is increasingly recognized as a critical strategy for addressing health system challenges in LMICs by fostering inclusive, context-specific solutions. A 2024 systematic review identified over 20 public health interventions across LMICs that successfully used coproduction or co-creation approaches, emphasizing the importance of local stakeholder involvement and contextual tailoring(xx). For example, participatory planning in Uganda and India improved hygiene and maternal health outcomes, respectively, by aligning interventions with community needs (xx). These models demonstrate that coproduction enhances trust, relevance, and sustainability, key to overcoming systemic barriers in under-resourced health systems(yy)  . |
