# Supplementary material for: Transforming multi-stakeholder engagement towards coproduction of optimized maternal, newborn, and child health and a resilient community health system in rural Ethiopia: A qualitative study
Source: PLoS One. 2025 Aug 26;20(8):e0330159. doi: 10.1371/journal.pone.0330159 (PMC12380333; doi:10.1371/journal.pone.0330159)
Supplement: Peer review history

# Peer review: Decision Letter 0

**DOI:** 10.1371/journal.pone.0330159.r001

**Authors:** Rachidatou Compaoré

7 Feb 2024

Transforming multi-stakeholder engagement towards coproduction of optimized maternal, newborn, and child health and a resilient community health system in rural Ethiopia: A qualitative study

PLOS ONE

Dear Dr. Gebremeskel,

Thank you for submitting your manuscript to PLOS ONE. After careful consideration, we feel that it has merit but does not fully meet PLOS ONE’s publication criteria as it currently stands. Therefore, we invite you to submit a revised version of the manuscript that addresses the points raised during the review process.

Please submit your revised manuscript by Mar 23 2024 11:59PM. If you will need more time than this to complete your revisions, please reply to this message or contact the journal office at plosone@plos.org . When you're ready to submit your revision, log on to https://www.editorialmanager.com/pone/ and select the 'Submissions Needing Revision' folder to locate your manuscript file.

Please include the following items when submitting your revised manuscript:

- A rebuttal letter that responds to each point raised by the academic editor and reviewer(s). You should upload this letter as a separate file labeled 'Response to Reviewers'.
- A marked-up copy of your manuscript that highlights changes made to the original version. You should upload this as a separate file labeled 'Revised Manuscript with Track Changes'.
- An unmarked version of your revised paper without tracked changes. You should upload this as a separate file labeled 'Manuscript'.
- In the abstract, situate the study in time, report the results so that they are more specific to what you found in your study and ensure that the recommendations made are based on the reported results.
  In the introduction, check the accuracy of the reported data
  In the method section,  
  In the results section, structure the results to give a common thread to the readers.
   Ensure that all abbreviations have been defined from the first use.
  Ensure that all references meet the recommendations made by the newspaper

If you would like to make changes to your financial disclosure, please include your updated statement in your cover letter. Guidelines for resubmitting your figure files are available below the reviewer comments at the end of this letter.

If applicable, we recommend that you deposit your laboratory protocols in protocols.io to enhance the reproducibility of your results. Protocols.io assigns your protocol its own identifier (DOI) so that it can be cited independently in the future. For instructions see: https://journals.plos.org/plosone/s/submission-guidelines#loc-laboratory-protocols . Additionally, PLOS ONE offers an option for publishing peer-reviewed Lab Protocol articles, which describe protocols hosted on protocols.io. Read more information on sharing protocols at https://plos.org/protocols?utm_medium=editorial-email&utm_source=authorletters&utm_campaign=protocols .

We look forward to receiving your revised manuscript.

Kind regards,

Rachidatou Compaoré, MD, MSc

Academic Editor

PLOS ONE

Journal Requirements:

When submitting your revision, we need you to address these additional requirements.

1. Please ensure that your manuscript meets PLOS ONE's style requirements, including those for file naming. The PLOS ONE style templates can be found at 

https://journals.plos.org/plosone/s/file?id=wjVg/PLOSOne_formatting_sample_main_body.pdf and 

https://journals.plos.org/plosone/s/file?id=ba62/PLOSOne_formatting_sample_title_authors_affiliations.pdf

2. Thank you for stating the following financial disclosure: 

   "NO"

At this time, please address the following queries:

a) Please clarify the sources of funding (financial or material support) for your study. List the grants or organizations that supported your study, including funding received from your institution. 

b) State what role the funders took in the study. If the funders had no role in your study, please state: “The funders had no role in study design, data collection and analysis, decision to publish, or preparation of the manuscript.”

c) If any authors received a salary from any of your funders, please state which authors and which funders.

d) If you did not receive any funding for this study, please state: “The authors received no specific funding for this work.”

Please include your amended statements within your cover letter; we will change the online submission form on your behalf.

3. We note that you have indicated that there are restrictions to data sharing for this study. PLOS only allows data to be available upon request if there are legal or ethical restrictions on sharing data publicly. For more information on unacceptable data access restrictions, please see http://journals.plos.org/plosone/s/data-availability#loc-unacceptable-data-access-restrictions. 

Before we proceed with your manuscript, please address the following prompts:

a) If there are ethical or legal restrictions on sharing a de-identified data set, please explain them in detail (e.g., data contain potentially identifying or sensitive patient information, data are owned by a third-party organization, etc.) and who has imposed them (e.g., a Research Ethics Committee or Institutional Review Board, etc.). Please also provide contact information for a data access committee, ethics committee, or other institutional body to which data requests may be sent.

b) If there are no restrictions, please upload the minimal anonymized data set necessary to replicate your study findings to a stable, public repository and provide us with the relevant URLs, DOIs, or accession numbers. For a list of recommended repositories, please see

https://journals.plos.org/plosone/s/recommended-repositories. You also have the option of uploading the data as Supporting Information files, but we would recommend depositing data directly to a data repository if possible.

We will update your Data Availability statement on your behalf to reflect the information you provide.

4. Please include captions for your Supporting Information files at the end of your manuscript, and update any in-text citations to match accordingly. Please see our Supporting Information guidelines for more information: http://journals.plos.org/plosone/s/supporting-information. 

5. Please review your reference list to ensure that it is complete and correct. If you have cited papers that have been retracted, please include the rationale for doing so in the manuscript text, or remove these references and replace them with relevant current references. Any changes to the reference list should be mentioned in the rebuttal letter that accompanies your revised manuscript. If you need to cite a retracted article, indicate the article’s retracted status in the References list and also include a citation and full reference for the retraction notice.

Additional Editor Comments:

- In the abstract, specify the period of study, report the results so that they are more specific to what you found in your study, and ensure that the recommendations made are based on the reported findings.
- In the introduction, check the accuracy of the reported data
- In the results section, structure the results to give a common thread to the readers and present the different themes in a balanced way
-  Ensure that all abbreviations have been defined from the first use.
- Ensure that all references meet the journal's recommendations for citations.

[Note: HTML markup is below. Please do not edit.]

Reviewers' comments:

Reviewer's Responses to Questions

**Comments to the Author**

1. Is the manuscript technically sound, and do the data support the conclusions?

Reviewer #1: Yes

Reviewer #2: Yes

**********

2. Has the statistical analysis been performed appropriately and rigorously?

Reviewer #1: Yes

Reviewer #2: Yes

**********

3. Have the authors made all data underlying the findings in their manuscript fully available?

The PLOS Data policy

Reviewer #1: Yes

Reviewer #2: Yes

**********

4. Is the manuscript presented in an intelligible fashion and written in standard English?

Reviewer #1: Yes

Reviewer #2: Yes

**********

Reviewer #1: Thank you for the study that highlights a growing interest and need to incorporate community voices and ideas in what affects their own health in an attempt to get communities to own and become responsible for their health. the article is generally well written, with a few suggestions to consider.

Please check that the numbers are correct on Introduction, page 4, paragraph 4 on the proportion of health finance.

In the results section, I suggest that the authors reduce text on the much longer themes and have a balance in the number of quotes presented across all themes.

Under Barriers, "the challenges of alignment", "lack of systemic coordination and patforms" and inadequate

coordination and implementation capacity" seem to refer to the same thing in their current presentation.

THe Abbreviation list is not complete, e.g. SSA not included

There a few typing errors. e.g. finding vs findings

Reference: there is not consistency in the reference list, stick to one style and and be consistent to what you choose. Some years missing, use of short or full names for journal names, use of month and date for some and not for others.

Reviewer #2: Abstract

In the abstract section-Methods: We conducted a qualitative case study – not recommended in research writing to use We/I – instead better - A qualitative case study was conducted …

Check and correct all – in the case of using abbreviation in the first place write the full word (abbreviation).

In the abstract the conclusion is not supported by the result stated?

Methods

In research setting – The authors wrote more detail about Ethiopia but focus on the study setting.

In this research, the nested nature of the case study encompasses two distinct populations including HEWs/CHWs (16 FGD participant), and subnational and national public health policy actors (12 KII participant).

The authors not include the community or service users – so lack triangulation in this aspect. If you can better inclued.

KII participants Key

Informants were recruited based on their known involvement in the policy process leading to the planning and the implementation of CHWs/ MNCH program.

The authors need specify the number from working at regional and ministry separately – because these are responsible for policy issues.

**********

what does this mean? ). If published, this will include your full peer review and any attached files.

If you choose “no”, your identity will remain anonymous but your review may still be made public.

**Do you want your identity to be public for this peer review?** For information about this choice, including consent withdrawal, please see our Privacy Policy

Reviewer #1: No

Reviewer #2: **Yes: ** Esubalew Tesfahun

**********

[NOTE: If reviewer comments were submitted as an attachment file, they will be attached to this email and accessible via the submission site. Please log into your account, locate the manuscript record, and check for the action link "View Attachments". If this link does not appear, there are no attachment files.]

While revising your submission, please upload your figure files to the Preflight Analysis and Conversion Engine (PACE) digital diagnostic tool, https://pacev2.apexcovantage.com/ . PACE helps ensure that figures meet PLOS requirements. To use PACE, you must first register as a user. Registration is free. Then, login and navigate to the UPLOAD tab, where you will find detailed instructions on how to use the tool. If you encounter any issues or have any questions when using PACE, please email PLOS at figures@plos.org

# Author comment: Author response to Decision Letter 1

**DOI:** 10.1371/journal.pone.0330159.r002

5 Mar 2024

Thank you Dr. Rachidatou Compaoré for reviewing and positive feedback on our manuscript. Please see attached document for your critical comments.

Attachment Submitted filename: Response to Reviewers.docx

# Peer review: Decision Letter 1

**DOI:** 10.1371/journal.pone.0330159.r003

**Authors:** Tatchalerm Sudhipongpracha

9 Apr 2025

Dear Dr. Gebremeskel,

Thank you for submitting your manuscript to PLOS ONE. After careful consideration, we feel that it has merit but does not fully meet PLOS ONE’s publication criteria as it currently stands. Therefore, we invite you to submit a revised version of the manuscript that addresses the points raised during the review process.

The reviewers have provided the following suggestions. Please make your best effort in addressing them.

Reviewer #3:

1. Addressing PLOS ONE’s open data policy by specifying whether anonymized transcripts or coding frameworks can be shared.

2. Expand the conclusion by providing more actionable recommendations on how policymakers and practitioners can implement the findings.

Reviewer #4:

Introduction (Pages 1-5)

o Page 5- I would like to see the figure of the final theoretical framework.

o The section could clarify how multi-stakeholder engagement differs from traditional public-private partnerships.

o Consider briefly discussing power dynamics (government vs. NGOs, local vs. international stakeholders).

Methods (Pages 5-8)

o It would be helpful to clarify why West Shewa Zone was selected.

o You said it was translated from Amharic and Oromia languages, how did you the translation consistency and inter-rater reliability? Consider discussing inter-coder reliability or alternative interpretations.

o Page 8 - Explain why data was manually coded instead of using qualitative software like NVivo. Provide more details on how data saturation was determined and why manual coding was preferred over software.

o Please add more detail on how themes were developed inductively/deductively would strengthen credibility. If it is both inductive and deductive, specify how themes emerged and how prior frameworks influenced coding.

o In the recent publications by Braun & Clarke (2021), emphasized the importance of clarifying the theoretical and philosophical assumptions underlying the chosen thematic analysis procedure. Clearly state epistemological and ontological assumptions. Is the study critical realist, constructivist, or positivist? Does it assume an objective reality (realism) or multiple realities (relativism)?

Results (Pages 9-15)

o Some quotes are long, consider summarizing or paraphrasing for clarity.

o The concept of "donor dependency" is discussed but could be linked to alternative funding solutions.

Discussion (Pages 16-19)

o More discussion on CHW mental health/workload challenges would strengthen practical implications.

o Provide examples of successful coproduction models from other LMICs for comparison.

Please submit your revised manuscript by May 24 2025 11:59PM. If you will need more time than this to complete your revisions, please reply to this message or contact the journal office at plosone@plos.org . When you're ready to submit your revision, log on to https://www.editorialmanager.com/pone/ and select the 'Submissions Needing Revision' folder to locate your manuscript file.

- A rebuttal letter that responds to each point raised by the academic editor and reviewer(s). You should upload this letter as a separate file labeled 'Response to Reviewers'.
- A marked-up copy of your manuscript that highlights changes made to the original version. You should upload this as a separate file labeled 'Revised Manuscript with Track Changes'.
- An unmarked version of your revised paper without tracked changes. You should upload this as a separate file labeled 'Manuscript'.

If applicable, we recommend that you deposit your laboratory protocols in protocols.io to enhance the reproducibility of your results. Protocols.io assigns your protocol its own identifier (DOI) so that it can be cited independently in the future. For instructions see: https://journals.plos.org/plosone/s/submission-guidelines#loc-laboratory-protocols . Additionally, PLOS ONE offers an option for publishing peer-reviewed Lab Protocol articles, which describe protocols hosted on protocols.io. Read more information on sharing protocols at https://plos.org/protocols?utm_medium=editorial-email&utm_source=authorletters&utm_campaign=protocols .

We look forward to receiving your revised manuscript.

Kind regards,

Tatchalerm Sudhipongpracha

Academic Editor

PLOS ONE

Journal Requirements:

Please review your reference list to ensure that it is complete and correct. If you have cited papers that have been retracted, please include the rationale for doing so in the manuscript text, or remove these references and replace them with relevant current references. Any changes to the reference list should be mentioned in the rebuttal letter that accompanies your revised manuscript. If you need to cite a retracted article, indicate the article’s retracted status in the References list and also include a citation and full reference for the retraction notice.

[Note: HTML markup is below. Please do not edit.]

Reviewers' comments:

Reviewer's Responses to Questions

**Comments to the Author**

Reviewer #2: All comments have been addressed

Reviewer #3: All comments have been addressed

Reviewer #4: (No Response)

**********

2. Is the manuscript technically sound, and do the data support the conclusions?

Reviewer #2: Yes

Reviewer #3: Yes

Reviewer #4: Yes

**********

3. Has the statistical analysis been performed appropriately and rigorously?

Reviewer #2: Yes

Reviewer #3: N/A

Reviewer #4: N/A

**********

4. Have the authors made all data underlying the findings in their manuscript fully available?

The PLOS Data policy

Reviewer #2: Yes

Reviewer #3: No

Reviewer #4: No

**********

5. Is the manuscript presented in an intelligible fashion and written in standard English?

Reviewer #2: Yes

Reviewer #3: Yes

Reviewer #4: Yes

**********

Reviewer #2: I see this revised manuscript and confirmed that all my comments have been addressed, no addtional comments.

Reviewer #3: The manuscript presents a timely study and addresses critical gaps in the literature regarding the intersection of multi-stakeholder collaboration and community health resilience. The authors provide a thorough background, theoretical framework, and methodology to support their research.

Regarding response to previous comments, the authors have adequately responded to prior concerns and incorporated relevant revisions.

The manuscript describes a technically sound qualitative study using appropriate data collection and analysis methods, with a well detailed thematic analysis. The findings are in line with the data presented, which further supports the study’s conclusions.

A statistical analysis is not applicable in this study as this is a qualitative study. However, the thematic coding and methodological rigor ensure validity.

Regarding data availability, although the data availability statement indicates restrictions on sharing interview transcripts due to ethical considerations. However, PLOS ONE has an open data policy that encourages data transparency. I would recommend that the authors specify if anonymized versions of transcripts or coding frameworks could be shared upon reasonable request.

Overall, the manuscript is well-organized, and the language is clear and intelligible. The study fills an important gap in understanding multi-stakeholder engagement for MNCH services in Ethiopia, and its findings have practical implications for improving stakeholder collaboration in community health systems. However, there are a few areas for improvement such as;

1. Addressing PLOS ONE’s open data policy by specifying whether anonymized transcripts or coding frameworks can be shared.

2. Expand the conclusion by providing more actionable recommendations on how policymakers and practitioners can implement the findings.

Reviewer #4: It is very important topic and the study addresses a critical public health issue, high maternal and child mortality in Ethiopia. The title clearly reflects the study's focus on multi-stakeholder engagement and coproduction in MNCH services.

Introduction (Pages 1-5)

o Page 5- I would like to see the figure of the final theoretical framework.

o The section could clarify how multi-stakeholder engagement differs from traditional public-private partnerships.

o Consider briefly discussing power dynamics (government vs. NGOs, local vs. international stakeholders).

Methods (Pages 5-8)

o It would be helpful to clarify why West Shewa Zone was selected.

o You said it was translated from Amharic and Oromia languages, how did you the translation consistency and inter-rater reliability? Consider discussing inter-coder reliability or alternative interpretations.

o Page 8 - Explain why data was manually coded instead of using qualitative software like NVivo. Provide more details on how data saturation was determined and why manual coding was preferred over software.

o Please add more detail on how themes were developed inductively/deductively would strengthen credibility. If it is both inductive and deductive, specify how themes emerged and how prior frameworks influenced coding.

o In the recent publications by Braun & Clarke (2021), emphasized the importance of clarifying the theoretical and philosophical assumptions underlying the chosen thematic analysis procedure. Clearly state epistemological and ontological assumptions. Is the study critical realist, constructivist, or positivist? Does it assume an objective reality (realism) or multiple realities (relativism)?

Results (Pages 9-15)

o Some quotes are long, consider summarizing or paraphrasing for clarity.

o The concept of "donor dependency" is discussed but could be linked to alternative funding solutions.

Discussion (Pages 16-19)

o More discussion on CHW mental health/workload challenges would strengthen practical implications.

o Provide examples of successful coproduction models from other LMICs for comparison.

**********

what does this mean? ). If published, this will include your full peer review and any attached files.

If you choose “no”, your identity will remain anonymous but your review may still be made public.

**Do you want your identity to be public for this peer review?** For information about this choice, including consent withdrawal, please see our Privacy Policy

Reviewer #2: **Yes: ** Esubalew Tesfahun

Reviewer #3: **Yes: ** Chibuzor Stella Amadi

Reviewer #4: **Yes: ** Muluken Basa

**********

[NOTE: If reviewer comments were submitted as an attachment file, they will be attached to this email and accessible via the submission site. Please log into your account, locate the manuscript record, and check for the action link "View Attachments". If this link does not appear, there are no attachment files.]

While revising your submission, please upload your figure files to the Preflight Analysis and Conversion Engine (PACE) digital diagnostic tool, https://pacev2.apexcovantage.com/ . PACE helps ensure that figures meet PLOS requirements. To use PACE, you must first register as a user. Registration is free. Then, login and navigate to the UPLOAD tab, where you will find detailed instructions on how to use the tool. If you encounter any issues or have any questions when using PACE, please email PLOS at figures@plos.org

# Author comment: Author response to Decision Letter 2

**DOI:** 10.1371/journal.pone.0330159.r004

27 May 2025

Please see attached documenet : Response

Attachment Submitted filename: Response to Reviwers-24 May.docx

# Peer review: Decision Letter 2

**DOI:** 10.1371/journal.pone.0330159.r005

**Authors:** Tatchalerm Sudhipongpracha

29 Jul 2025

Transforming multi-stakeholder engagement towards coproduction of optimized maternal, newborn, and child health and a resilient community health system in rural Ethiopia: A qualitative study

PONE-D-23-23186R2

Dear Dr. Gebremeskel,

We’re pleased to inform you that your manuscript has been judged scientifically suitable for publication and will be formally accepted for publication once it meets all outstanding technical requirements.

Within one week, you’ll receive an e-mail detailing the required amendments. When these have been addressed, you’ll receive a formal acceptance letter and your manuscript will be scheduled for publication.

An invoice will be generated when your article is formally accepted. Please note, if your institution has a publishing partnership with PLOS and your article meets the relevant criteria, all or part of your publication costs will be covered. Please make sure your user information is up-to-date by logging into Editorial Manager at Editorial Manager®  and clicking the ‘Update My Information' link at the top of the page. For questions related to billing, please contact billing support .

If your institution or institutions have a press office, please notify them about your upcoming paper to help maximize its impact. If they’ll be preparing press materials, please inform our press team as soon as possible -- no later than 48 hours after receiving the formal acceptance. Your manuscript will remain under strict press embargo until 2 pm Eastern Time on the date of publication. For more information, please contact onepress@plos.org.

Kind regards,

Tatchalerm Sudhipongpracha

Academic Editor

PLOS ONE

Additional Editor Comments (optional):

Reviewers' comments:

Reviewer's Responses to Questions

**Comments to the Author**

Reviewer #2: All comments have been addressed

Reviewer #4: All comments have been addressed

**********

2. Is the manuscript technically sound, and do the data support the conclusions?

Reviewer #2: Yes

Reviewer #4: Yes

**********

3. Has the statistical analysis been performed appropriately and rigorously?

Reviewer #2: Yes

Reviewer #4: Yes

**********

4. Have the authors made all data underlying the findings in their manuscript fully available?

The PLOS Data policy

Reviewer #2: Yes

Reviewer #4: Yes

**********

5. Is the manuscript presented in an intelligible fashion and written in standard English?

Reviewer #2: Yes

Reviewer #4: Yes

**********

Reviewer #2: All comments have been addresses in this version, no addtional comments and concern in this revised version.

Reviewer #4: To the Editors of PLOS ONE,

Thank you for the opportunity to review the revised version of this important and timely manuscript. I appreciate the authors’ thoughtful and thorough responses to the previous round of feedback. After reviewing the revised manuscript and the detailed point-by-point responses, I am pleased to confirm that the authors have fully addressed all prior comments and recommendations.

Given the substantial improvements and the robust nature of the research, I am satisfied with the current version of the manuscript and recommend it for publication in PLOS ONE without further revision.

Thank you again for the opportunity to contribute to the review process.

Warm regards,

**********

what does this mean? ). If published, this will include your full peer review and any attached files.

If you choose “no”, your identity will remain anonymous but your review may still be made public.

**Do you want your identity to be public for this peer review?** For information about this choice, including consent withdrawal, please see our Privacy Policy

Reviewer #2: **Yes: ** Esubalew Tesfahun

Reviewer #4: **Yes: ** Muluken Basa

**********

# Editor report: Acceptance letter

**DOI:** 10.1371/journal.pone.0330159.r006

**Authors:** Tatchalerm Sudhipongpracha

PONE-D-23-23186R2

PLOS ONE

Dear Dr. Gebremeskel,

I'm pleased to inform you that your manuscript has been deemed suitable for publication in PLOS ONE. Congratulations! Your manuscript is now being handed over to our production team.

At this stage, our production department will prepare your paper for publication. This includes ensuring the following:

* All references, tables, and figures are properly cited

* All relevant supporting information is included in the manuscript submission,

* There are no issues that prevent the paper from being properly typeset

You will receive further instructions from the production team, including instructions on how to review your proof when it is ready. Please keep in mind that we are working through a large volume of accepted articles, so please give us a few days to review your paper and let you know the next and final steps.

Lastly, if your institution or institutions have a press office, please let them know about your upcoming paper now to help maximize its impact. If they'll be preparing press materials, please inform our press team within the next 48 hours. Your manuscript will remain under strict press embargo until 2 pm Eastern Time on the date of publication. For more information, please contact onepress@plos.org.

You will receive an invoice from PLOS for your publication fee after your manuscript has reached the completed accept phase. If you receive an email requesting payment before acceptance or for any other service, this may be a phishing scheme. Learn how to identify phishing emails and protect your accounts at https://explore.plos.org/phishing.

If we can help with anything else, please email us at customercare@plos.org.

Thank you for submitting your work to PLOS ONE and supporting open access.

Kind regards,

PLOS ONE Editorial Office Staff

on behalf of

Dr. Tatchalerm Sudhipongpracha

Academic Editor

PLOS ONE
